# Berufliche und Angehörigenpflege leisten – Scoping-Review zu Erfahrungen doppelt herausgeforderter Pflegender

**DOI:** 10.1007/s00391-024-02382-5

**Published:** 2024-11-26

**Authors:** Nicole Ruppert, Martina Roes

**Affiliations:** 1 Fakultät für Gesundheit, Department für Pflegewissenschaft, Universität Witten-Herdecke, Witten, Deutschland; 2 Arbeitsgruppe Implementierungswissenschaft & Personzentrierte Demenzversorgung, Deutsches Zentrum für Neurogenerative Erkrankungen e. V. (DZNE), Witten, Deutschland

**Keywords:** Doppelte Pflegeaufgabe, Formelle Pflege, Informelle Pflege, Doppelrolle, Rollenkonflikt, Nurse-family member, Formal care, Informal care, Dual role, Role conflict

## Abstract

**Hintergrund:**

Professionell Pflegende, die zudem Angehörige pflegen, hier benannt als doppelt herausgeforderte Pflegende (dhP), leisten doppelte Pflegearbeit. Unklar ist, ob sie im Versorgungskontext andere Herausforderungen zu bewältigen haben als pflegende Angehörige ohne professionellen Hintergrund.

**Ziel:**

Dieser Beitrag soll ein besseres Verständnis sowie einen aktuellen Überblick geben, wie professionell Pflegende ihre doppelte Pflegeaufgabe erleben und mit welchen Herausforderungen sie konfrontiert werden.

**Methoden:**

Die systematische Literaturrecherche erfolgte in 11 Fachdatenbanken sowie per Schneeballsystem. Eingeschlossen wurden 32 deutsch- und englischsprachige Artikel, veröffentlicht im Zeitraum von Januar 1995 bis Oktober 2023.

**Ergebnisse:**

Es wurden 6 Themenkomplexe identifiziert, die das Erleben und die Herausforderungen von dhP fokussieren: *emotionale und persönliche Aspekte, Anforderungen und Erwartungen, persönliche Bedürfnisse, Rollenfindung, pflegerisches Fachwissen* und *Interaktion mit Fachpersonen des Gesundheitswesens*.

**Schlussfolgerung:**

Die Herausforderungen für dhP sind vielschichtig. Die Ergebnisse zeigen, dass v. a. die Rollenfindung im Pflegearrangement herausfordernd ist, da die berufliche und die private Pflegerolle eng miteinander verwoben sind. Die Präferenz für eine dieser Rollen variiert nach Persönlichkeit, Art der Beziehungen oder Pflegesituation. Oftmals wird diese Problematik vom Umfeld nicht wahrgenommen bzw. respektiert, was vorhandenes Belastungserleben verstärken kann. Andererseits nutzen dhP ihre beruflichen und privaten Erfahrungen, um im jeweils anderen Kontext gute Pflege- und Sorgearbeit zu leisten.

**Zusatzmaterial online:**

Zusätzliche Informationen sind in der Online-Version dieses Artikels (10.1007/s00391-024-02382-5) enthalten.

## Abstract (en)

**Background:**

Professional nurses who also care for relatives are called double duty caregivers and provide double care work. It is not obvious whether this means they have to cope with other challenges than caring relatives without a professional background.

**Objective:**

This article aims to gain a better understanding and an overview of how double duty caregivers experience their dual role and the challenges they face.

**Methods:**

The systematic literature search was conducted in 11 specialized databases as well as via a snowball system. Included in the study were 32 articles published in German and English between January 1995 and October 2023.

**Results:**

A total of 6 topic complexes were identified that deal with the experience and challenges of double duty caregivers: emotional and personal aspects, demands and expectations, personal needs, role identification, nursing expertise and interaction with professionals in the healthcare system.

**Conclusion:**

The challenges for double duty caregivers are complex. The results show that finding their own role in the nursing arrangement is particularly challenging as the professional and private nursing roles are closely intertwined. The preference for one of these roles varies depending on the personality, type of relationship or care situation. This problem is often not recognized or respected by the environment, which can reinforce existing feelings of stress. On the other hand, double duty caregivers use their professional and private experience to provide good nursing and care in the respective other context.

## Hintergrund

Im Jahr 2015 wurde der *Unabhängige Beirat für die Vereinbarkeit von Beruf und Pflege* vom Bundesministerium für Familie, Senioren, Frauen und Jugend eingesetzt. Dieser betont, dass Sorgearbeit sowohl eine gesamtgesellschaftliche Aufgabe als auch eine individuelle Entscheidung für oder gegen die Übernahme von Angehörigenpflege ist [23]. Diese Entscheidung gilt es zu respektieren [23, 24]. Im Bericht zum Gender Care Gap wird betont, dass ohne Sorgearbeit gesellschaftliches Leben oder wirtschaftliches Wachstum nicht möglich seien [3]. Frauen leisten dabei mehr unbezahlte häusliche Sorgearbeit als Männer und stehen dem von Frauen dominierten pflegerischen Arbeitsmarkt deshalb seltener zur Verfügung [3]. Doch gibt es professionell Pflegende, die zudem Angehörige pflegen, sog. Double Duty Caregiver oder hier doppelt herausgeforderte Pflegende (dhP) genannt. Deren genaue Anzahl ist unbekannt, verschiedene Quellen gehen von ca. 8–20 % in Deutschland aus [6, 7].

Die *Konzertierte Aktion Pflege* [4] initiierte diverse Unterstützungsmaßnahmen, um die Übernahme von beruflicher Pflege und Angehörigenpflege zu ermöglichen, Personal in den Pflegeeinrichtungen zu halten sowie dem Fachpersonenmangel entgegenzuwirken. Laut GKV-Spitzenverband [12] haben im Jahr 2022, Stand April 2023, 81 Krankenhäuser finanzielle Leistungen nach § 4 Abs. 8a Krankenhausentgeltgesetz (KHEntgG) abgerufen, um Maßnahmen zur Unterstützung von Mitarbeiter:innen mit privater Pflegeverantwortung, wie beispielsweise flexible Arbeitszeiten, umzusetzen. Fraglich ist, ob diese Formen der Unterstützung für dhP angemessen und ausreichend sind. Dazu ist es hilfreich zu wissen, welche Unterstützung dhP parallel zu ihrer Expertise benötigen bzw. erwarten, und welche Herausforderungen dafür ursächlich sind.

Aus diesem Grund wurde, im Rahmen einer Dissertation zum Thema, im ersten Schritt dieses Scoping-Review erstellt, mit dem Ziel, einen Überblick über den derzeitigen Stand der Diskussion und ein besseres Verständnis für das Erleben und die Herausforderungen von professionell Pflegenden, die zudem Angehörige pflegen, zu erlangen. Die übergeordnete Forschungsfrage lautet: Wie erleben beruflich Pflegende ihre Situation als pflegende Angehörige? Ergänzende Fragen sind: 1) Mit welchen Herausforderungen werden dhP konfrontiert, und wie bewältigen sie diese? 2) Welche Vor- oder Nachteile sehen dhP in ihrer beruflichen Qualifikation, und wie, glauben sie, wirkt sie sich auf ihre Rolle und Aufgaben als pflegende Angehörige aus?

## Methoden

Zur Beantwortung der auf Basis des PCC-Frameworks [8] entwickelten Forschungsfragen wurde ein Scoping-Review durchgeführt, orientiert am Konzept PRISMA-ScR [8]. Die Literaturrecherche wurde in den Fachdatenbanken CareLit®, CINAHL, Cochrane, GeroLit, Medline, ProQuest, PsychInfo®, PSYNDEX, PubMed, Scopus, Web of Science™ sowie per Schneeballsystem durchgeführt.

Die Generierung der Forschungsfragen sowie der Suchwörter erfolgte auf Grundlage der mittels PCC-Schema [8] erstellten Ein- und Ausschlusskriterien (s. Zusatzmaterial online).

**Abb. 1:**
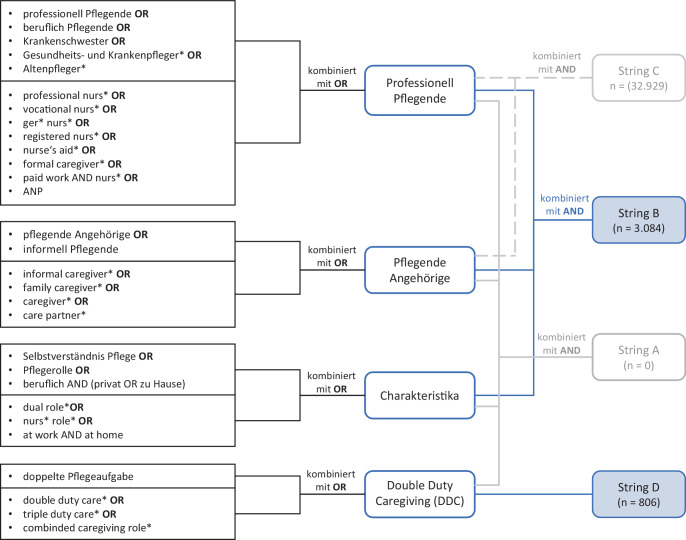
Strategie der Literaturrecherche

Eingeschlossen wurden im Zeitraum von Januar 1995 bis September 2020 veröffentlichte englisch- und deutschsprachige Artikel, unabhängig vom Studiendesign. Der Recherchezeitpunkt ab 1995 wurde gewählt, da in diesem Jahr in Deutschland das Sozialgesetzbuch XI und somit spezielle Unterstützungsleistungen für pflegende Angehörige in Kraft traten. Ausgeschlossen wurden Kongressbeiträge und Websites. Die englischen und deutschen Suchwörter wurden von N.R. und M.R. in enger Abstimmung festgelegt und den Oberbegriffen *professionell Pflegende, pflegende Angehörige, Charakteristika* sowie *Double Duty Caregiving *zugeordnet. Nach PPC-Schema steht die Kombination der Oberbegriffe *professionell Pflegende* und *pflegende Angehörige* für die *Population* der doppelt Pflegenden, die Benennung der beruflichen Qualifikationen sowie die familiären Verbindungen berücksichtigen den *Context*. Sowohl *Charakteristika* als auch *Double Duty Caregiving *stehen für das *Concept*. Eine Übersicht der Suchbegriffe und -strategie für die Datenbankrecherche findet sich in Abb. 1. Sofern möglich, wurde sowohl nach Mesh-Terms als auch Stichworten in Titeln und Abstracts gesucht (N.R.). Für den Zeitraum Oktober 2020 bis Oktober 2023 wurde eine Nachrecherche entsprechend der ersten Suchstrategie vorgenommen (N.R.). Die Angaben in den Abb. 1 und 2 berücksichtigen den gesamten Recherchezeitraum.

**Abb. 2:**
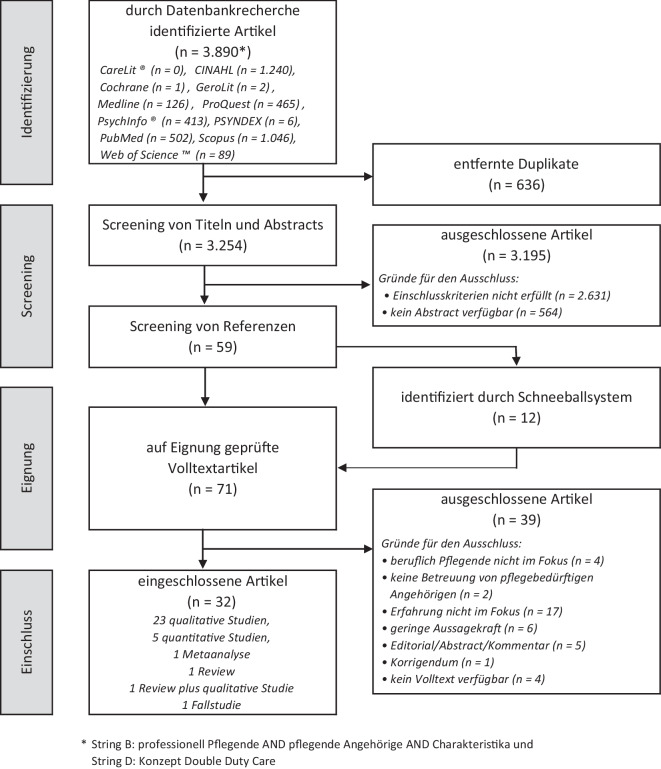
Flowchart Such- und Auswahlprozess nach PRISMA-Statement [8]

Die Kombination der Ergebnisse aller vier Oberbegriffe mit dem Bool-Operator AND (String A) ergab keine Treffer. Da der Oberbegriff *Double Duty Caregiving* und dessen Suchwörter aber das Konzept konkret benennen, wurde dieser von den *Charakteristika* getrennt als String (D) mitaufgenommen (N.R., M.R). Die Kombination der Oberbegriffe *Professionell Pflegende* AND *Pflegende Angehörige *(String C) hingegen, gewählt um Quellen zu finden, die das Konzept anders benennen, hatte mit annähernd 33.000 Quellen eine zu hohe, unspezifische Trefferquote (Abb. 1). Aus diesem Grund wurden nach gemeinsamer Beratung (N.R., M.R.) nur die Ergebnisse der Strings B und D (Abb. 1) der weiteren Eignungsprüfung unterzogen. Die Auswahl der identifizierten Volltexte sowie Absprachen bei Problemstellungen fanden iterativ zwischen N.R. und M.R. statt.

## Ergebnisse

Nach Entfernung der 636 Duplikate wurden die verbliebenen 3254 identifizierten Artikel von Suchstring B und D (Abb. 1) einem Screening unter Berücksichtigung der Ein- und Ausschlusskriterien unterzogen (N.R.). Die Sichtung der Titel und Abstracts sowie eine Schneeballsuche ergab 71 Referenzen, die zur Eignungsprüfung im Volltext gescreent wurden (N.R., M.R.). Für die Verwaltung der Quellen sowie der Volltexte wurde die Literaturdatenbank Citavi 6 genutzt. Die Artikelauswahl (N.R.) und die spätere Datenextraktion (N.R.) erfolgten entsprechend der JBI-Methodologie [8]. Insgesamt eigneten sich 32 Artikel (23 qualitative und 5 quantitative Studien, 1 Metaanalyse, 1 Review, 1 Review plus qualitative Studie, 1 Fallstudie) (Abb. 2) für die abschließende Volltextanalyse (N.R., M.R.). Die Artikel stammen aus Kanada (8), den USA (8), Australien (7), England (2), den Niederlanden (2), der Schweiz (2), Neuseeland (1), Portugal (1) und Schweden (1). Es konnte keine Studie aus Deutschland identifiziert werden, die den Einschlusskriterien entsprach. Einige der eingeschlossenen Studien stammen aus demselben Forschungskontext, sodass auf Basis von 5 Studien 12 Artikel veröffentlicht wurden. Ein Artikel verbindet die Ergebnisse eines Scoping-Reviews und einer qualitativen Studie (s. Zusatzmaterial online).

Die Analyse der Artikel erfolgte in Anlehnung an die strukturierte qualitative Inhaltsanalyse nach Kuckartz und Rädiker [18] (vorrangig N.R.). Der Analyseprozess erfolgte induktiv, um alle thematisierten Aspekte der Artikel zu erfassen bzw. zusammenzufassen (N.R.). Aus den herausgearbeiteten Codes wurden sechs Kategorien gebildet (N.R., M.R.), im Folgenden als Themenkomplexe bezeichnet: *A) emotionale und persönliche Aspekte, B) Anforderungen und Erwartungen, C) persönliche Bedürfnisse, D) Rollenfindung, E) pflegerisches Fachwissen *und *F) Interaktion mit Fachpersonen des Gesundheitswesens.* In diesem Artikel werden nur die am häufigsten thematisierten Ergebnisse dargestellt. Diese wurden stellvertretend für alle eingeschlossenen Studien mit den Quellen belegt, die mehrere Themenkomplexe aufgreifen und im Rahmen unterschiedlicher Forschungskontexte entstanden. Dazu zählen nur qualitative Studien, in denen pro Studie maximal 32 Pflegefachpersonen interviewt wurden. Entsprechende Ergebnisse werden auch in den hier nicht aufgeführten Studien beschrieben (quantitativ, große Stichproben mit 257 bis 1406 Probanden, zusätzlich zu Pflegefachpersonen auch Pflegeassistentinnen einbeziehend bzw. ausschließlich Letztere fokussierend, durchgeführt in den Niederlanden, Neuseeland und der Schweiz). Im Zusatzmaterial online finden sich ein Gesamtüberblick der Studien sowie der identifizierten Themenkomplexe, eine inhaltliche Übersicht (Stichproben, Ergebnisse etc.) aller eingeschlossenen Studien, Reviews sowie die dazugehörigen Literaturangaben.

### A) Emotionale und persönliche Aspekte

Der Fokus liegt hier vorwiegend auf den negativen Erfahrungen durch die Übernahme von sowohl beruflicher als auch privater Pflegeaufgabe. Interviewte äußern Gefühle, nicht als Kind wahrgenommen zu werden [26], aufgrund ihres beruflichen Hintergrundes immer auf Abruf und im Dienst zu sein sowie der Pflege nicht entkommen zu können [22, 25]. Weitere Studien zeigen, dass dhP teils Wut und Frustration erleben, wenn beispielsweise Familienmitglieder ihre Expertise und Hilfe ablehnen [26] oder sie den Eindruck haben, Dienstleister würden nicht korrekt handeln [13, 26]. Auch Gefühle von Unzulänglichkeit und Ohnmacht, ausgelöst durch untätiges Danebensitzen [25], sowie Schuldgefühle bei fehlender erforderlicher Expertise oder nach falsch getroffenen pflegebezogenen Entscheidungen [25] werden thematisiert. Belastend sind beispielsweise das Wissen, was kommen wird, oder die Isolation durch den Rückzug von der Familie, um nicht als negativ und überreagierend zu gelten [19, 25, 26]. Studienergebnisse zeigen auch, dass dhP trotz hoher informeller Pflegebelastung eine positive Einstellung zu ihrem Beruf haben und ihre persönlichen Erfahrungen in den beruflichen Kontext, z. B. in der Angehörigenarbeit und Unterstützung von Kollegen, einbringen [26].

### B) Anforderungen und Erwartungen

Die Ergebnisse der Studien zeigen, dass sich die familiären Rollen und die damit verbundenen Anforderungen an dhP durch ihre Profession verändern. Dies löst z. T. ambivalente Gefühle aus. Viele dhP sind sich aufgrund ihres Fachwissens sicher, das Richtige für ihre Angehörigen zu tun, während andere sich von ihren Familien unter Druck gesetzt fühlen, da diese eine umfassende Expertise in pflegerischen und medizinischen Belangen voraussetzen [2, 5, 9, 10, 13, 19, 22, 26]. Konkret wird erwartet, dass dhP medizinische Informationen übersetzen, die Pflege übernehmen oder Entscheidungen für den zu pflegenden Angehörigen treffen [9, 11, 19]. Der Druck wird verstärkt, wenn sie das Gefühl haben, gedrängt zu werden, mehr Verantwortung zu übernehmen als sie können oder wollen [25]. Des Weiteren kommen auch in diesen Zusammenhängen Gefühle der Unzulänglichkeit, Verlegenheit, Frustration und Angst auf, wenn sie den Erwartungen des pflegebedürftigen Angehörigen, der Familie und ihren eigenen Ansprüchen an die Pflege nicht entsprechen bzw. diese nicht erfüllen können [13, 21, 26]. Um weiterhin adäquat handeln und für die Familie da sein zu können, werden diese Gefühle oft unterdrückt [5, 10, 21] bzw. Grenzen gesetzt [10] oder ausgehandelt [22].

### C) Persönliche Bedürfnisse

Die identifizierten Studien zeigen, dass dhP sich kontinuierlich dafür einsetzen, eine qualitativ hochwertige Pflege ihrer Angehörigen sicherzustellen [9, 25]. Sie haben hohe Ansprüche an die eigene Sorgearbeit und übertragen dies auch auf andere Leistungserbringer [26]. Persönlich wünschen sich dhP mehr Unterstützung von Fachpersonen während der Begleitung von sterbenden Angehörigen, insbesondere bei fehlender Erfahrung [9, 10]. Einige Studien zeigen, dass sich emotional einsame oder isolierte dhP nach Entlastung durch und Austausch mit Gleichgesinnten sehnen [9, 10, 19]. Darüber hinaus wünschen sie sich, ihre Bedürfnisse äußern zu können, sowie deren Wahrnehmung und Berücksichtigung durch beteiligte Fachpersonen [9, 19]. Dazu gehören beispielsweise umfassende, individuell auf ihre spezifischen Bedürfnisse zugeschnittene Informationen und Unterstützung, die sie nicht erhalten, wenn z. B. Ärzt:innen ihnen gegenüber äußern, ein mehr an Informationen sei nicht notwendig [9–11].

### D) Rollenfindung

Nach Studienlage ist es ein zentrales Anliegen der dhP, ihre Rolle im Versorgungsarrangement zwischen pflegebedürftigen Angehörigen, der gesamten Familie, Freunden sowie dem beruflichen Umfeld zu finden [1, 2, 5, 9–11, 15, 19–22, 25, 26]. Dazu müssen sie sich klar werden, ob sie primär pflegende Angehörige oder Pflegefachpersonen sind bzw. sein wollen. Die Rollen können im Verlauf wechseln, beeinflusst u. a. von Setting, interagierenden Personen und Intensität der Einbindung in die Sorgearbeit [1, 2, 5, 9–11, 15, 19–22, 25, 26]. Wie Studienergebnisse zeigen, sind für dhP beide Rollen untrennbar miteinander verbunden, doch nutzen sie ihre Profession, um beispielsweise effektive Pflege und Unterstützung zu leisten [10, 11, 13, 21, 26]. Doppelt herausgeforderte Pflegende können erst entspannen, ihre eigenen Gefühle zulassen und sich ganz der Angehörigenrolle widmen, wenn sie Vertrauen zum betreuenden Personal und in deren geleistete Sorgearbeit gefasst und/oder diese sie dazu aufgefordert haben [11, 21, 26]*.* Eine Studie fokussierte die Rolle von Männern. Die Analyse ergab u. a., dass diese, aufgrund vorherrschender gesellschaftlicher Normen, meist selbst entscheiden können, wie stark sie sich engagieren und übernehmen bevorzugt Aufgaben der Koordination und Organisation [1]. Weibliche dhP hingegen, so eine andere Studie, erhalten aufgrund dieser gesellschaftlichen Normen weniger Unterstützung und Anerkennung [25]. Der Wunsch männlicher dhP, selbst pflegerisch aktiv zu werden, stößt aufgrund des überwiegend geltenden Rollenbildes eher auf allgemeines Unverständnis in den Familien [1].

### E) Pflegerisches Fachwissen

Laut Studienlage erachten dhP ihre pflegerische Expertise überwiegend als Vorteil für die Bewältigung der informellen Pflege. Ihre Kenntnisse ermöglichen, detaillierte Informationen zu erhalten, für Angehörige zu übersetzen und diese zu beraten, Maßnahmen zu planen und einzufordern, Prozesse zu koordinieren, die Qualität von Informationen, geleisteter Pflege, Testergebnissen und den Gesundheitsstatus zu beurteilen sowie durch das Gesundheitssystem zu navigieren [2, 5, 10, 19, 20, 22, 26]. Zum Schutz ihrer pflegebedürftigen Angehörigen agieren sie als Fürsprecher, Vermittler oder Überwacher und greifen in Risikosituationen sowie bei unterlassener oder inadäquater Pflege auch praktisch ein [5, 10, 11, 13, 15, 20–22, 26]. Dabei achten sie stets darauf, ihre eigene Profession nicht zu denunzieren [15]. Weitere Studien zeigen, dass das Fachwissen, wie in bereits genannten Bezügen, emotional belasten kann und u. a. Angst, Besorgnis oder Stress auslöst, z. B. bei Erkennen von Gefahren oder Lücken im Versorgungssystem [2, 5, 10, 20, 21, 25]. Zudem haben dhP z. T. Probleme, Entscheidungen von Angehörigen zu akzeptieren bzw. auszuhalten, wenn diese nicht ihrem Fachwissen entsprechen [5]. Die eigene emotionale Verfassung sowie der meist fachfremde Kontext der Angehörigenpflege können verunsichern und das Gefühl vermitteln, das eigene Fachwissen reiche nicht aus [11, 15, 26]. Des Weiteren beschreiben dhP, ohne die ihnen im beruflichen Umfeld verfügbaren Ressourcen keine häusliche Pflege entsprechend ihres Verständnisses leisten zu können [22]. Zudem äußern sie Unbehagen, sich auf Pflegearbeiten einzulassen, für die sich nicht qualifiziert genug fühlen [22, 25]. Viele dhP nutzen in solchen Situationen ihre pflegerische Expertise, um derartige Aufgaben abzulehnen und Grenzen zu setzen [13, 22].

### F) Interaktion mit Fachpersonen des Gesundheitswesens

Eine Studie verdeutlichte, wie hilfreich es für die betroffene Zielgruppe ist, die Sprache der professionellen Dienstleister zu sprechen [2]. Dabei müssen sie jedoch häufig klarstellen, dass sie als pflegende Angehörige und nicht als Pflegefachperson Kontakt suchen [2]. Doppelt herausgeforderte Pflegende bemühen sich, ein gutes Verhältnis zum fremden professionellen Team aufzubauen, um respektiert und einbezogen zu werden, damit sie die bestmögliche Versorgung ihrer Angehörigen garantieren und ggf. konstruktive Kritik äußern können [10, 15, 20, 21]. Sie möchten als *gute Angehörige* wahrgenommen werden, angepasst, unkritisch und nicht störend, um die Zusammenarbeit mit den professionellen Dienstleistern nicht zu gefährden [10, 19]. Der Aufbau einer Beziehung zum Versorgungsteam und somit der Erhalt von Respekt, Informationen oder Rat von Kollegen, fällt dhP am eigenen Arbeitsort leichter [21]. Doch vermeiden es dhP, sich Rat einzuholen, um nicht als überfordert wahrgenommen zu werden [2]. Weitere Studien zeigen, dass dhP sich im Kontext fremder Einrichtungen permanent fragen, ob sie ihre Profession offenbaren sollen [11, 15, 22]. Verheimlicht wird dies z. B., um den eigenen Status und den der Angehörigen zu wahren, nicht anders beurteilt und behandelt zu werden, Angehörige vor unprofessioneller und gefährlicher Pflege zu bewahren oder nicht als schwierig zu gelten [11, 15, 19, 22]. Andere dhP, die ihre Profession offenbarten, berichteten, besser informiert, beachtet und respektiert zu werden [26].

## Diskussion

Die Ergebnisse der Literaturrecherche zeigen, für dhP ist nicht nur trotz, sondern auch aufgrund ihrer pflegespezifischen Expertise die Übernahme von Angehörigenpflege weder einfach noch selbstverständlich. Die emotionale Nähe zu ihren pflegebedürftigen Angehörigen belastet sie stärker als die Pflege im beruflichen Kontext. Hinzu kommen die, wie auch Köhler et al. [17] aufzeigen, familiären, nicht immer selbst gewählten Rollen, die zusätzlich ängstigen und Druck aufbauen können [2, 5, 9, 10, 13, 19, 20, 22, 25, 26]. Andererseits ist in diesen Fällen die von Kim et al. [16] beschriebene *professionelle Distanz* hilfreich, um trotz aller Emotionalität die Situation objektiv zu erfassen und adäquat zu handeln.

Herausfordernd für dhP ist es, allen an der Pflege beteiligten Personen zu verdeutlichen, dass sie trotz ihrer Doppelrolle eine von diesen präferieren, je nach Persönlichkeit, Beziehung zur pflegebedürftigen Person oder Situation [1, 2, 5, 9–11, 15, 19–22, 25, 26]. Dies geht mit unterschiedlichen Bedürfnissen einher. So wünschen sich dhP in ihrer Rolle als pflegende Angehörige z. B. umfassende Informationen oder Begleitung [9–11] und als Pflegefachperson als vollwertiges Mitglied im Versorgungsteam aufgenommen zu werden [10, 15, 20, 21]. Was sie erleben, ist, dass weder ihre Bedürfnisse wahrgenommen noch spezielle oder individuelle bedarfsgerechte Unterstützungsangebote gemacht werden. Dies gilt sowohl für das private Umfeld als auch für den Umgang mit Beschäftigten im Gesundheitswesen, insbesondere, wenn diese von der pflegerischen Expertise wissen. Statt zu unterstützen, scheinen Letztere dazu zu neigen, Tätigkeiten und Verantwortung abzugeben oder sich distanziert bis ablehnend zu zeigen [11, 15]. Dieses Verhalten gegenüber der eigenen Berufsgruppe erstaunt und trägt vermutlich dazu bei, dass dhP sich permanent fragen, ob sie sich als Pflegefachperson offenbaren sollen [11, 15, 22].

Während die Berufstätigkeit anderen pflegenden Angehörigen eine Auszeit von der informellen Pflege ermöglicht [14], können dhP keinen Abstand gewinnen, sondern fühlen sich oft, als wären sie ununterbrochen im Dienst [22, 25]. Die meisten dhP betonen aber, ihren Beruf zu lieben und ihn nicht aufgeben zu wollen [26]. Dies sollte Arbeitgeber veranlassen, Beschäftigte bei ihren Vereinbarkeitsbemühungen zu unterstützen, mit dem Effekt, dass der gesamtgesellschaftliche Auftrag, wie eingangs beschrieben [23], erfüllt und gleichzeitig dem Personalmangel im Pflegebereich entgegengewirkt werden kann. In diesem Zusammenhang wurde insbesondere die Rolle der pflegenden Männer bisher nicht bzw. nur unzureichend berücksichtigt. Lediglich eine Studie von 2012 [1] befasste sich mit der Situation von pflegenden Männern. Dieser Umstand und das augenscheinliche Festhalten an traditionellen Rollenbildern [1], wie sie eingangs auch im Zusammenhang mit dem Gender Care Gap [3] thematisiert wurden, bilden eine Grundlage für weitere Forschungen in diesem Kontext.

## Limitationen

Die Definitionen und Bezeichnungen für dhP sind vielfältig, und somit ist es möglich, dass nicht alle in die Suchstrategie integriert wurden. Da keine entsprechenden Beiträge aus Deutschland gefunden wurden, ist eine Übertragung auf den deutschen Raum schwierig. Zudem wurden weniger Studien durchgeführt, als die Anzahl der einbezogenen Artikel vermuten lässt, da zu einigen Studien mehrfach Artikel veröffentlicht wurden (s. Zusatzmaterial online). Ferner weisen einige Autoren auf eine begrenzte Aussagekraft bzw. Übertragbarkeit der Ergebnisse ihrer Studien hin. Deshalb können die vorliegenden Ergebnisse nicht grundsätzlich generalisiert werden.

## Schlussfolgerung

Ziel dieses Scoping-Reviews ist es, ein besseres Verständnis für das Erleben und die Herausforderungen von professionell Pflegenden, die zudem Angehörige pflegen, zu erlangen. Die Herausforderungen für dhP sind vielschichtig und unterscheiden sich in mehrfacher Hinsicht von denen anderer pflegender Angehöriger. Die eingangs beschriebenen Unterstützungsmaßnahmen, wie Anpassungen von Arbeitszeitmodellen oder Familienpflegezeit [4, 12], sind ein Schritt in die richtige Richtung, aber weiterführende Maßnahmen sind notwendig. Diese sollten sowohl körperliche, emotionale und fachliche Unterstützung bieten, dabei die Expertise der dhP berücksichtigen und auf dieser aufbauen. Des Weiteren ist es notwendig, dass von Seiten der Beschäftigten im Gesundheitswesen die Wahrnehmung der Bedürfnisse der betroffenen Zielgruppe geschärft, auf deren besonderen Bedarfe eingegangen sowie der eigene Umgang mit dhP reflektiert werden, damit Interaktionen weniger belastend sind. Das in den Studien identifizierte negative Verhalten von Beschäftigten im Gesundheitswesen gegenüber dhP [11, 15] weist auf weiteren Forschungsbedarf hin, ebenso wie die Analyse der Sichtweisen auf bzw. Erfahrungen mit dhP. Diese Erkenntnisse wären z. B. für die Weiterentwicklung von Unterstützungs- und Schulungskonzepten sowohl innerhalb als auch außerhalb von Einrichtungen des Gesundheitswesens hilfreich. Gelingt es, doppelt Pflegende derart zu unterstützen, dass sie die doppelte Pflegeaufgabe gut bewältigen, leisten sie einen doppelten Beitrag zur Sicherstellung informeller und formeller Pflege [3, 23], den es besonders zu würdigen gilt.

## Supplementary Information

Das Supplementary enthält 1. Informationen zu den Ein- und Ausschlusskriterien der Artikelauswahl, 2. eine Übersicht der Zuordnung der eingeschlossenen Artikel zu den identifizierten Themenkomplexen, 3. eine tabellarische inhaltliche Übersicht der eingeschlossenen Studien (Methoden, Stichprobe etc.) und 4. vollständiges Literaturverzeichnis aller eingeschlossenen Studien und zugehöriger Basisartikel
