# Erratum zu: Berufliche und Angehörigenpflege leisten – Scoping-Review zu Erfahrungen doppelt herausgeforderter Pflegender

**DOI:** 10.1007/s00391-025-02419-3

**Published:** 2025-02-17

**Authors:** Nicole Ruppert, Martina Roes

**Affiliations:** 1 Fakultät für Gesundheit, Department für Pflegewissenschaft, Universität Witten-Herdecke, Witten, Deutschland; 2 Arbeitsgruppe Implementierungswissenschaft & Personzentrierte Demenzversorgung, Deutsches Zentrum für Neurogenerative Erkrankungen e. V. (DZNE), Witten, Deutschland

**Erratum zu:**

**Z Gerontol Geriat 2024**

10.1007/s00391-024-02382-5

Der Artikel „Berufliche und Angehörigenpflege leisten – Scoping-Review zu Erfahrungen doppelt herausgeforderter Pflegender“, geschrieben von Nicole Ruppert und Martina Roes, wurde ursprünglich unter exklusiver Lizenz von „The Author(s), under exclusive licence to Springer Medizin Verlag GmbH, ein Teil von Springer Nature“ veröffentlicht. Als Ergebnis der anschließenden Entscheidung den Artikel im Open-Access-Modell zu veröffentlichen, wurde der Copyright-Vermerk des Artikels am 28. Januar 2025 in © „The Author(s) 2024“ geändert.

Dieser Artikel wird unter der Creative Commons Namensnennung 4.0 International Lizenz veröffentlicht, welche die Nutzung, Vervielfältigung, Bearbeitung, Verbreitung und Wiedergabe in jeglichem Medium und Format erlaubt, sofern Sie den/die ursprünglichen Autor(en) und die Quelle ordnungsgemäß nennen, einen Link zur Creative Commons Lizenz beifügen und angeben, ob Änderungen vorgenommen wurden.

Die in diesem Artikel enthaltenen Bilder und sonstiges Drittmaterial unterliegen ebenfalls der genannten Creative Commons Lizenz, sofern sich aus der Abbildungslegende nichts anderes ergibt. Sofern das betreffende Material nicht unter der genannten Creative Commons Lizenz steht und die betreffende Handlung nicht nach gesetzlichen Vorschriften erlaubt ist, ist für die oben aufgeführten Weiterverwendungen des Materials die Einwilligung des jeweiligen Rechteinhabers einzuholen.

Weitere Details zur Lizenz entnehmen Sie bitte der Lizenzinformation auf http://creativecommons.org/licenses/by/4.0/deed.de

