# Supplementary material for: Providing professional and family care—Scoping review on the experiences of double duty caregivers
Source: Z Gerontol Geriatr. 2024 Nov 26;58(4):317–23. [Article in German] doi: 10.1007/s00391-024-02382-5 (PMC12238111; doi:10.1007/s00391-024-02382-5)
Supplement: Supplementary file 1 — Das Supplementary enthält 1. Informationen zu den Ein- und Ausschlusskriterien der Artikelauswahl, 2. eine Übersicht der Zuordnung der eingeschlossenen Artikel zu den identifizierten Themenkomplexen, 3. eine tabellarische inhaltliche Übersicht der eingeschlossenen Studien (Methoden, Stichprobe etc.) und 4. vollständiges Literaturverzeichnis aller eingeschlossenen Studien und zugehöriger Basisartikel [file 391_2024_2382_MOESM1_ESM.docx]

**Tabelle 1: Einschluss- und Ausschlusskriterien nach PCC-Framework^[[1]](#footnote-1)^**

|  | Einschlusskriterien | Ausschlusskriterien |
| --- | --- | --- |
| P - Population | - professionell Pflegende im Gesundheitswesen (keine organisatorischen Einschränkungen, auch freiberuflich oder ehemals Pflegende) - Qualifikation: Pflegefachpersonen mit Ausbildung (einjährig oder vergleichbar, je nach Land) - die gleichzeitig pflegebedürftige Angehörige zu Hause versorgen | - Beschäftigte im Gesundheitswesen, die einer anderen Professionen als der Pflege angehören, wie beispielsweise Ärzte/Ärztinnen, Arzthelfer:innen, Sozialarbeiter:innen, Ehrenamtler:innen, Auszubildende |
| C - Concept | - Doppelte Pflegeaufgabe/Double Duty Caregiving = Betreuung und Pflege von alten und kranken Menschen sowohl zu Hause, als auch im Beruf - Dreifache Pflegeaufgabe/Triple Duty Caregiving = Betreuung und Pflege von eigenen Klein-/Kindern sowie pflegebedürftigen Angehörigen, als auch Betreuung und Versorgung von alten und kranken im Beruf - Oder: zusätzlich zu Double Duty Caregiving Betreuung von Kindern im eigenen Haushalt - Selbstverständnis der Pflege - Rolle | - Nur Betreuung eigener, gesunder Kinder |
| C - Context | - alle Pflegepersonen, die in der Akut-, Langzeit und ambulanten Pflege arbeiten, da die gesammelten Erfahrungen in den diversen Settings unterschiedliche Einflüsse auf die Bewältigung der privaten Pflegesituation/-aufgabe haben könnten - häusliche Pflege, unabhängig davon, ob die pflegebedürftigen Personen und pflegenden Angehörigen im selben oder in getrennten Haushalten leben | - Versorgung der pflegebedürftigen Angehörigen finden in Einrichtungen der stationären Langzeitpflege statt |

**Tabelle 2: Zuordnung der eingeschlossenen Studien zu den fokussierten Themenkomplexen.**

| Quellen | Themenkomplex  A B C D E F | | | | | |
| --- | --- | --- | --- | --- | --- | --- |
| Anjos et al. 2012^1^ | ✓ |  |  | ✓ |  |  |
| Baumblatt et al. 2022^2^ |  |  |  |  |  | ✓ |
| Boumans und Dorant 2014^2^ | ✓ |  |  |  |  |  |
| Brindley 2018^3^ |  | ✓ |  |  | ✓ | ✓ |
| Carlsson et al. 2016^1^ |  |  |  | ✓ | ✓ |  |
| Cicchelli und McLeod 2012^1^ |  | ✓ |  | ✓ | ✓ |  |
| Clendon und Walker 2017^1^ |  |  |  |  |  | ✓ |
| DePasquale et al. 2016b^2^ | ✓ |  |  |  |  |  |
| DePasquale et al. 2018a^2^ | ✓ |  |  |  |  |  |
| Detaille et al. 2020^1,4^ | ✓ |  |  |  |  | ✓ |
| Fouto und Partington 2016^1^ | ✓ | ✓ | ✓ | ✓ |  |  |
| Giles und Hall 2014^4^ |  | ✓ | ✓ | ✓ | ✓ | ✓ |
| Giles und Williamson 2015^1^ | ✓ | ✓ | ✓ | ✓ | ✓ | ✓ |
| Hansen 2016^1^ | ✓ | ✓ |  | ✓ | ✓ |  |
| Häusler et al. 2017^2^ | ✓ |  |  |  |  |  |
| Jähnke et al. 2017^1^ |  |  |  | ✓ | ✓ | ✓ |
| Jones 2020^1^ |  |  |  | ✓ | ✓ | ✓ |
| Klages et al. 2020a^1^ |  |  |  | ✓ |  | ✓ |
| Klages et al. 2020b^1^ |  |  |  | ✓ |  |  |
| Klages et al. 2020c^1^ | ✓ |  |  |  |  |  |
| Mills und Aubeeluck 2006^1^ |  | ✓ | ✓ | ✓ | ✓ | ✓ |
| Quinney et al. 2018a^1^ |  |  |  | ✓ | ✓ |  |
| Quinney et al. 2018b^1^ | ✓ | ✓ |  | ✓ | ✓ | ✓ |
| Salmond 2011^1^ | ✓ | ✓ |  | ✓ | ✓ | ✓ |
| Santerre-Theil et al. 2022^1^ | ✓ | ✓ |  | ✓ |  |  |
| St-Amant et al. 2014^1^ | ✓ | ✓ |  | ✓ | ✓ | ✓ |
| Ward-Griffin 2004^1^ |  |  |  | ✓ |  |  |
| Ward-Griffin et al. 2005^1^ |  |  |  | ✓ |  |  |
| Ward-Griffin et al. 2011^1^ | ✓ | ✓ | ✓ | ✓ | ✓ |  |
| Ward-Griffin et al. 2015^1^ |  | ✓ |  | ✓ | ✓ |  |
| Wilson und Ardoin 2013^5^ | ✓ |  |  | ✓ | ✓ |  |
| Wohlgemuth et al. 2015^1^ | ✓ | ✓ | ✓ | ✓ | ✓ | ✓ |
| *Artikeltyp: ^1^qualitative Studie, ^2^quantitative Studie, ^3^Metaanalyse, ^4^Review, ^5^Fallstudie* | | | | | | |

**Tab. 3: Übersicht der eingeschlossenen Studien**

| Autor(en),  Land, TK^[[2]](#footnote-2)^, (Primärstudie/Projekt) | Design,  Methode | Stichprobe | Limitationen | Schlüsselergebnisse der Studien,  nächste Schritte und Wissens-/  Forschungslücken | Schlüsselergebnisse mit Bezug  zu den Forschungsfragen |
| --- | --- | --- | --- | --- | --- |
| Anjos et al., 2012  Kanada  A, D  Sekundäranalyse zur Studie von Ward-Griffin et al., 2010 | Design  Qualitative Sekundäranalyse  Methode   - Telefoninterviews - Sekundäranalyse von Transkripten und Feldnotizen   Datenanalyse  Fokussiertes Kodieren | Gesamtstichprobe (n=28)  Geschlecht  Männlich (n=28)  Profession  Pflegefachperson  Arbeitsort/Funktion   - Krankenhaus - Gemeinde/ambulante Pflege - Führungspersonen - Mitarbeiter in Abteilungen mit hohem Pflegeaufwand   Lebenssituation  Keine Angaben  Pflegebeziehung   - Überwiegend Schwieger-/Eltern - 1 Angehörige (61%) - 2 Angehörige (28%) - 3 Angehörige (11%) | Keine Angaben | Schlüsselergebnisse   - Drei Determinanten der Pflege im Kontext geschlechtsspezifischer Erwartungen und Ausnahmeregelungen (hier männliche DDCs): (1) familiäre Verpflichtungen und Beziehungen, (2) pflegerische Kenntnisse und Fähigkeiten sowie (3) Zugang zu Ressourcen - Erwartungen und Ausnahmeregelungen sind miteinander verwoben und treten gleichzeitig auf. Männliche DDCs tendieren dazu, dies zu ihrem Vorteil zu nutzen - Männliche DDCs erfahren sowohl geschlechtsspezifische Vor- als auch Nachteile, z.B. die Wahl zu haben, welche Tätigkeiten übernommen und welche delegiert werden und/oder negative Auswirkungen auf die eigene Gesundheit   Nächste Schritte/Forschungslücken   - Analyse der Auswirkungen der Doppelbelastung mit besonderem Fokus auf geschlechtsspezifische Machverhältnisse - Untersuchungen zu den geschlechtsspezifischen Erwartungen und Ausnahmen von männlichen DDC verschiedener sexuelle Orientierungen notwendig - Theorieentwicklung zur Dekonstruktion von mit der Pflege verbundenen Geschlechterideologien | - Männliche DDC machen die Erfahrung, dass die geschlechtsspezifischen Erwartungen an ihre Rolle in der privaten Pflegesituation nicht mit den eigenen, durch ihre Profession geprägte, übereinstimmen - Männliche DDCs agieren eher Geschlecht stereotypisch, d. h., dass sie sich eher aus der direkten Pflege ziehen und Aufgaben delegieren. Möchten männliche DDC sich mehr in der direkten Pflege engagieren, machen sie die Erfahrung, dass dies auf Ablehnung stößt, da es nicht dem vorherrschendem geschlechtsspezifischen Rollenbild entspricht |
| Baumblatt et al., 2022  USA  F | Design  Quantitative  Querschnittstudie  Methode  Fragebogen  Einschlusskriterien   - Informelle Pflegeerfahrung - Voraussetzung bei Pflegepersonal - Keine Voraussetzung bei Führungskräften   Datenanalyse   - Deskriptive Analyse der statistischen Daten - Quantitative-qualitative Matrix mit exemplarischen Textantworten aus offenen Fragen | Gesamtstichprobe (n=257)  Geschlecht   - Pflegefachfrau (93,5%) - Pflegefachmann (6,5%) - weibliche Führungspersonen (87,3%) - männliche Führungspersonen (12,7%)   Profession   - Pflegefachpersonen (n=206) - Führungspersonen in Gesundheitsorganisationen (n=51)   Arbeitsort/Funktion  Keine Angaben  Lebenssituation  Keine Angaben  Pflegebeziehung   - Eltern - Partner:innen - Kinder - Großeltern - Geschwister - Cousin:en - Freunde | Nicht übertragbar auf andere Länder aufgrund der unterschiedlichen pflege- und sozialrechtlichen Unterstützungsstrukturen   - Keine Definition der Dauer und Intensität der geleisteten privaten Pflege - Fehlende Reliabilität der Konstrukte, da für jedes nur ein Item vorgesehen war - Geringe Anzahl an Führungskräften | Schlüsselergebnisse   - Meist findet berufliche Unterstützung in Form von Familienpflegezeit statt, was jedoch nicht ausreichend ist, da diese nur für DDC gilt, die ausreichend Überstunden angesammelt haben und sich in einer bestimmten Pflegekonstellation befinden - dies führt zu sozialer Ungleichheit, da sie nicht von DDC in Teilzeit oder ohne festes Angestelltenverhältnis genutzt werden kann - DDC ist ein Risikofaktor für Burnout - Fehlende Unterstützung am Arbeitsplatz beeinflusst die Intention, den Arbeitsplatz zu wechseln   Nächste Schritte/Forschungslücken   - Auswirkungen unterschiedlicher Dauer und Intensität der privaten Pflege analysieren - Ethische Dilemmata von DDC während einer Pandemie untersuchen - Wirtschaftliche Kosten (bspw. Lohnausfall, Krankenversicherungsansprüche) und damit korrespondierende Nachteile analysieren - Beschäftigungsentscheidungen (bspw. Teilzeitpflege, Reisepflege) untersuchen. - Auswirkungen der informellen Pflege auf pflegerische Organisationen und deren Teams besser verstehen, um geeignete Dienstleistungen anbieten zu können | - DDC wünschen sich trotz ihres klinischen Fachwissens Unterstützung bei der Koordination, Beratung sowie Aus- und Weiterbildung - DDC erleben, dass Unterstützung und Verständnis für die private Pflege mit der Zeit abnehmen, aufgrund der Fehleinschätzung, dass es sich vor allem um akute und kurzzeitige Pflegebedürfnisse handelt, was in der Regel nicht der Fall ist. Dies kann zu Mitleidsermüdung und der Unterstellung führen, DDC würden die Situation ausnutzen. - DDC wünschen sich weitere Unterstützung als nur das Angebot der Familienauszeit, da nicht von allen nutzbar - DDC neigen dazu, bei fehlender sozialer Unterstützung das Arbeitsverhältnis aufzugeben (gilt für beide Gruppen) - Da sich DDC auf das Wohlergehen ihrer Angehörigen konzentrieren, nehmen sie negative Auswirkungen der doppelten Pflegeaufgabe auf die eigene Gesundheit nicht wahr |
| Boumans & Dorant, 2014  Niederlande  A | Design  Quantitative Querschnittstudie  Definierte Gruppen der informellen Pflege   - Hochintensive informelle Pflege ≥8 Stunden/Woche (n=48) - Informelle Pflege mit geringer Intensität ≤8 Stunden/Woche (n=45) ohne informelle Pflegeaufgabe (n=235)   Methode  Digitaler Fragebogen  Datenanalyse  Deskriptive Analyse | Gesamtstichprobe (n=328)  Geschlecht  *Frauenanteil pro Gruppe:*   - Hochintensive informelle Pflege 85,4% - Geringe informelle Pflege 95,6% - Ohne informelle Pflege 93,6%   Professionen   - Pflegefachperson und Pflegehelfer:in (92,2%) - Paramedizinische Bereiche (2,5%), z. B. Physiotherapeut:in, Ergotherapeut:in, Logopäd:in, Psycholog:in, Sozialarbeiter:in, Ernährungsberater:in, Seelsorger:in, Ärzt:in im Pflegeheim - Funktionen in der spezialisierten häuslichen Pflege (7,3%)   Arbeitsort/Funktion   - Krankenhaus - Pflegeheim   Lebenssituation  Keine Angaben  Pflegebeziehung   - Partner - Kind - Geschwister - (Schwieger-)Eltern - Freund:in - Nachbar:in - andere (Enkelsohn, Schwägerin, Großeltern) | - Durch das Querschnittsdesign keine kausalen Zusammenhänge herstellbar - Mögliche Beeinträchtigung der internen und externen Validität durch niedrige Rücklaufquote - Nutzung von Online-Befragung in der Urlaubszeit - Sample wahrscheinlich nicht repräsentativ - Kein validiertes Assessment verwendet | Schlüsselergebnisse   - DDC mit hochintensiver Pflegeaufgabe empfinden es signifikant schwerer, informelle und formelle Pflege miteinander zu vereinbaren - DDC haben auf der Arbeit mehr Probleme durch plötzliche Unterbrechungen, eine schlechtere Gesundheit, einen höheren Erholungsbedarf und fühlen sich emotional ausgebrannter als Pflegende mit geringer oder ohne private Pflegeaufgabe - DDC haben das Risiko Überlastungssymptome zu entwickeln und dann den Arbeitsplatz oder den Beruf zu verlassen   Nächste Schritte/Forschungslücken   - Entwicklung langfristiger Lösungen auf organisatorischer und gesetzlicher Ebene, um DDC im Beruf zu halten, wie familienfreundliche Maßnahmen am Arbeitsplatz, Pflegezahlungen und Pflegeurlaub | - DDCs mit hochintensiver informeller Pflegeaufgabe fühlen sich weniger in der Lage beiden Pflegeaufgaben gerecht zu werden, sind höheren Belastungen ausgesetzt als Pflegende mit geringer oder keiner informeller Pflegeaufgabe, was zu höherem Präsentismus, jedoch nicht zu höheren Fehlzeiten führt -> gehen häufiger krank zur Arbeit - DDC sind dennoch ihrer Arbeit gegenüber positiv eingestellt = keine Unterschiede in der Arbeitszufriedenheit und Motivation/Engagement im Vergleich zu Pflegenden ohne informelle Pflegeaufgabe - Die häufigsten informellen Pflegeaufgaben sind Koordination und Begleitung (hohe Intensität) |
| Carlsson et al., 2016  Schweden  D, E | Design   - Qualitative Studie - Phänomenografie   Methode  Halbstrukturierte Interviews  Datenanalyse  Phänomenografische Methode | Gesamtstichprobe (n=18)  Geschlecht   - Weiblich (n=16) - Männlich (n=2)   Professionen   - Pflegefachpersonen (n=9) - Ärzt:innen (n=2) - Sozialarbeiter:innen (n=2) - Hilfspfleger:in (n=1) - Hebamme (n=1) - Ergotherapeut:in (n=1) - Röntgenassistent:in (n=1) - Biomedizintechniker:in (n=1)   Mit mindestens ein Jahr Berufserfahrung  Arbeitsort/Funktion  Krankenhaus  Lebenssituation  Keine informelle Pflegebeziehung vor der Krankenhauseinweisung  Pflegebeziehung   - Ehepartner:in - Tochter, Sohn - Mutter - Schwiegervater, Großmutter, Enkelin | - Mögliche abweichende Ergebnisse in anderen Kulturbereichen und Kontexten - Rekrutierung über Aushänge in drei Krankenhäusern - Nur zwei männliche Teilnehmer - Nur drei Krankhäuser derselben Gesundheitsorganisation in einer Region beteiligt | Schlüsselergebnisse  DDC haben vier Auffassungen von ihrer Rolle als Angehörige von Patienten im Krankenhaus, die in hierarchischer Beziehung zueinanderstehen, ausgehend vom Grad der Involviertheit in die Pflege:   - *informierter Begleiter (informed bystander)* - sie wollen, dass ihnen zugehört wird, sie relevante Informationen erhalten und sie so ein gewisses Maß an Kontrolle erhalten - *Überwacher* *(supervisor)* - sie überwachen den medizinischen Status, Untersuchungsergebnisse sowie die pflegerische Versorgung, ohne sich einzumischen - *Fürsprecher (advocate)* - sie überwachen ebenso den medizinischen Status und die pflegerische Versorgung, instruieren aber ihre Angehörigen, was sie fragen oder einfordern sollen, beziehungsweise übernehmen dies selbst (RN n=4) - *Pflegende (carer)* - sie übernehmen die Überwachung und Kommunikation mit dem Stationspersonal sowie die Pflege bei unangemessener, unsicherer oder unterlassener Versorgung (RN n=2)   Nächste Schritte/Forschungslücken  Forschungsbedarf zu:   - Rollenkonflikten - Ambivalenz bezüglich Beziehungsaufbau und ‚Eingelassenwerden‘ in das Stationsteam - Erfahrungen, zur Pflege eines Angehörigen gezwungen zu sein. | - DDC erleben mehrere Aspekte des Rollenverständnisses, während ihre Angehörigen im Krankenhaus liegen. - *Überwacher, Fürsprecher* und *Pflegende* erleben einen Doppelrollenkonflikt, der sich aus der Loyalität für das eigene Familienmitglied und der Loyalität gegenüber der eignen Berufsgruppe ergibt. - Die Rollen der *Überwacher* und *Fürsprecher* zeigen, dass DDC zum Teil keine Pflege übernehmen können oder wollen - Es löst ambivalente Gefühle aus zu überlegen, ob sie sich offenbaren sollen oder nicht - Pflegefachfrauen nehmen eine aktivere Rolle ein (*Fürsprecher* (n=4), *Pflegende* (n=2)). |
| Cicchelli & McLeod, 2012  Kanada  B, D, E | Design   - qualitative Studie - hermeneutische Phänomenologie   Methode halbstrukturierte Interviews  Datenanalyse   - Interpretative Memos - thematische Analyse | Gesamtstichprobe (n=5)  Geschlecht  Weiblich (n=5)  Profession  Pflegefachperson  Arbeitsort/Funktion  Keine Angabe  Lebenssituation  Selber Haushalt oder in der Nähe wohnend  Pflegebeziehung   - Schwieger-/Eltern - Tante - alle mit einer fortgeschrittenen onkologischen Erkrankung | Kleine Stichprobe | Schlüsselergebnisse  *Zentrales Thema (S. 54):*   - *‘in der Mitte gefangen sein‚ (‚being caught in the middle‚*) - die Grenzen zwischen beruflicher und privater Pflege sind fließend und werden permanent neu verhandelt   *Unterthemen (S. 54):*   - Ausbalancierung beruflicher und persönlicher Grenzen - Besitz privilegierter Informationen - Erleben des Gesundheitssystems aus der Perspektive eines Pflegebedürftigen - Miterleben der Krankheit - Entscheidungsfindung für ihr Familienmitglied - Flexibilität am Arbeitsplatz als hilfreich erleben   Nächste Schritte/Forschungslücken  Keine Angaben | - DDC haben durch ihr Fachwissen besondere Erwartungen an die eigenen Leistungen. Sie wissen was kommt und versuchen sich und die Familie auf die nächsten Schritte vorzubereiten. Schwierig ist nur zu entscheiden, wie viele Informationen wie und wann weitergegeben werden. - DDC fühlen sich zwar verpflichtet, die Pflege zu übernehmen, sind aber auch zufrieden und stolz, wenn sie gut pflegen und Herausforderungen meistern. - Normen und Erwartungen an Pflegende leiten DDC, so dass sie ihre eigenen Bedürfnisse nicht wahrnehmen - DDC fühlen sich und ihre eigenen Bedürfnisse von Fachpersonen ignoriert - Die Erwerbstätigkeit ist gut für kurze Pausen von der häuslichen Pflege |
| Clendon & Walker, 2017  Neuseeland  F | Design   - Qualitative Studie - Explorativ, deskriptives Design   Methode   - Halbstrukturierte Einzelinterviews (n=11) - Halbstrukturierte Paar-Interviews (n=1) - Zwei halbstrukturierte Fokusgruppen (n=5, n=10)   Datenanalyse  Allgemeine induktive Vorgehensweise | Gesamtstichprobe (n=28)  Geschlecht  Unklar  Profession  Pflegefachperson  Arbeitsort/Funktion   - Krankenhaus (n=20) - Ambulante Pflege (n=4) - Psychiatrische Pflege (n=4)     Lebenssituation  Keine Angaben  Pflegebeziehungen   - Kinder (n=20) - Kinder und Eltern (n=4) - Pflegebedürftige ältere Erwachsene (n=4) - Kinder mit speziellen Betreuungsbedarf (n=11) | - Die Form der Rekrutierung kann ein Bias verursacht haben, da sich nur die stärker Betroffenen gemeldet haben könnten oder die am stärksten Betroffenen schon aus dem Dienst ausgeschieden waren - Fragliche Übertragbarkeit durch die kleine Stichprobe | Schlüsselergebnisse  *Kern- und die Subthemen (S. 95ff):*  1. *Jonglieren -* Gleichgewicht zwischen den Anforderungen finden und halten:  a) Familie an erster Stelle  b) Wahlmöglichkeit - zwischen Kindern und Karriere  c) Bedeutung der Arbeit - als Ausgleich und Unterstützung, persönlicher Wunsch oder Notwendigkeit  d) Schuldgefühle - nicht da zu sein, wo man gebraucht wird  2. *Persönliche Auswirkungen*:  a) Finanziell  b) Auf das Selbst - u.a. auf Gesundheit und Aktivitäten  c) Auf die Familie  3. *Umweltbezogene Faktoren*:  a) Der ‚verständnisvolle Chef‘  b) Unterstützung am Arbeitsplatz, z.B. durch Tagespflegeplätze  c) Flexibilität - der Arbeitszeiten  d) Urlaub - um Pflege zu leisten  Nächste Schritte/Forschungslücken  Keine Angaben | - Viele DDC vermeiden es, sich zu offenbaren, aus Angst dies könnte als ‚Nichtbewältigung‘ der Situation ausgelegt werden oder sich negativ auf künftige Arbeits- und Beförderungsaussichten auswirken - Auch Pflegemanager:innen sind betroffen und sind durch ihre Führungsposition doppelt isoliert |
| DePasquale et al., 2016  USA  A  Sekundäranalyse der Daten der Studie "Work, Family and Health Network" von Bray et. al. 2013 | Design  Quantitative Sekundäranalyse  Methode  Assessments zu Stress, Arbeit-Familien-Konflikt und Partnerbeziehung  Definierte Gruppen der informellen Pflege   - Keine (n=498) - Kinder (DDCC) (n=475) - Ältere Erwachsene (DDAC) (n=228) - Kinder und ältere Erwachsene (TDC) (n=196)   Datenanalyse   - Varianz-Tests - Multiple lineare Regressionsanalyse - ANCOVA | Gesamtstichprobe (n=1399)  Geschlecht  Weiblich (n=1399)    Professionen   - Pflegehelferin (67%) - Pflegefachperson (28%)   Arbeitsort/Funktion  Stationäre Langzeitpflege  Lebenssituation  Keine Angaben  Pflegebeziehung  Keine Angaben | - Korrelations- und Querschnittsdesign lässt keine Kausalität zu - Ergebnisse nicht auf andere Berufsgruppen im Gesundheitswesen übertragbar - Alle Teilnehmerinnen arbeiten für dasselbe Unternehmen - Nicht alle Kovarianten mit Einfluss auf das Stresserleben konnten untersucht werden - Möglicher Bass durch unbeobachtete Variablen | Schlüsselergebnisse   - Das Stresserleben bei DDAC and TDC ist höher als bei den anderen Gruppen. - Der wahrgenommene und psychologische Stress bei DDAC und TDC ist höher als in den anderen Gruppen, die sich aber kaum unterscheiden.   Nächste Schritte/Forschungslücken   - Arbeitsplatzanpassungen an die neuen Herausforderungen, um DDC und TDC zu halten - Forschung zu Prävalenz, Arten und Auswirkungen von DDC und TDC unter Frauen und Männern, in unterschiedlichen Berufen des Gesundheitswesens | - Partnerschaftliche Unterstützung ist wichtig für DDC und TDC. Fehlt diese Unterstützung, da sie Singles sind, muss diese von anderer Seite, z.B. dem Arbeitgeber, geleistet werden. - Bei bestehender informeller Unterstützung am Arbeitsplatz fürchten DDC evtl. Strafen, bei Inanspruchnahme oder Überbeanspruchung der Angebote - Sowohl DDC als auch TDC erleben höhere familiäre Belastungen, größere psychische Erschöpfung und höheren Stress und unterscheiden sich dabei kaum - TDC erleben darüber hinaus eine größere arbeitsbezogene Belastung als DDC |
| DePasquale et al., 2018  USA  A  Sekundäranalyse der Daten der Studie "Work, Family and Health Network" von Bray et. al. 2013 | Design  Quantitative Sekundäranalyse  Methode  Computer-unterstützte persönliche Interviews, Nutzung verschiedener Assessments  Definierte Gruppen der informellen Pflege   - Keine (n=342) - Kinder (DDCC) (n=330) - Erwachsene (DDAC) (n=160) - Kinder und Erwachsene (TDC) (n=140)   Datenanalyse  Deskriptiv | Gesamtstichprobe  (n=972)  Geschlecht  Weiblich (n=972)  Profession  Pflegeassistentin (n=972)  Arbeitsort/Funktion  Pflegeheim  Lebenssituation  Keine Angaben  Pflegebeziehung  Nur unterschieden nach Kindern oder Erwachsenen, nicht weiter ausdifferenziert | - Nur Querschnittsanalyse - Nur auf Mitarbeiterinnen in Pflegeheimen der USA beschränkt – keine Verallgemeinerung und Übertragbarkeit möglich - Sekundäranalyse von Daten, die für andere Forschungsfragen erhoben wurden | Schlüsselergebnisse   - TDC berichten höhere emotionale Erschöpfung, weniger Arbeitszufriedenheit und fühlen sich stärker verpflichtet, der Arbeit willen ‘Familienopfer‘ zu bringen, als nur am Arbeitsplatz tätige Personen. - Es wird vermutet, dass sich das Arbeitsumfeld (Setting) unterschiedlich auf dem Empfinden auswirkt. - Eine größere wahrgenommene Angemessenheit der Familienzeit reduzierte die emotionale Erschöpfung und die Fluktuationsabsichten der TDC. Auch kehrte sich die Wahrnehmung der TDC, hinsichtlich des Arbeitsklimas ‘Familienopfer‘ zu bringen, um.   Nächste Schritte/Forschungslücken   - Längsschnittstudien zur Untersuchung der Dynamik der Zusammenhänge - Erweiterte Studien mit differenzierteren Messgrößen, wie: Betreuungsstunden, Dauer der Betreuung, Anzahl, Art und Intensität der Betreuungsanforderungen oder -aktivitäten, Alter, Gesundheitszustand, Verhaltensprobleme, Beziehung zu doppelt und dreifach pflegenden Personen - Untersuchung, welche anderen psychologischen, beruflichen und familiären Effekte Einfluss auf die Arbeitsbelastung nehmen - Qualitativer Erhebungen, welche Faktoren am Arbeitsplatz die Familienzeit begünstigen oder reduzieren | - Das Setting, in dem Pflegende arbeiten, nimmt Einfluss auf das Erleben von Stress, Arbeitsbelastung etc. - TDC berichten eine größere Arbeitsunzufriedenheit als die anderen Gruppen - Wenn TDC die Zeit für die Familie als angemessen empfinden, reduzieren sich emotionale Erschöpfung und Fluktuationsabsichten sowie die Wahrnehmung, für die Arbeit ‚Familienopfer‘ bringen zu müssen - Es konnte ein Zusammenhang festgestellt werden, zwischen dem Empfinden der Angemessenheit der Familienzeit und dem Empfinden der Belastungen am Arbeitsplatz - Eine angemessene Familienzeit ist eine wichtige psychologische Ressource für DDC und TDC und sie sind darauf bedacht, diese zu erhalten, zu bewahren und zu schützen. |
| Detaille et al., 2020  Niederlande  F  Zweiter Teil im Artikel von Detaille et al., 2020 | Design  Qualitative Studie  Methode  Fokusgruppen mit Themenleitfaden (n=2)  Datenanalyse  Reflexive thematische Analyse | Gesamtstichprobe (n=17)  Geschlecht  Weiblich (n=17)  Profession  Pflegefachperson  Arbeitsort/Funktion  Altenpflegeeinrichtung  Lebenssituation  Keine Angaben  Pflegebeziehung  Keine Angaben | - Kleine Stichprobe - Keine männlichen Teilnehmer - Möglicher Bias durch Teilnahme interessierter und engagierter Personen (trotz Schichtarbeit, privater Einbindung etc.) | Schlüsselergebnisse   - Da DDC über Fachkenntnisse und Erfahrungen verfügen, sind sie die einzigen in der Familie, die diese Aufgabe übernehmen können - Aufgrund von Übermüdung und mangelnder Erholung in der Woche finden es DDC schwierig, die doppelten Pflegeaufgaben zu bewältigen. - Es sollten Selbstmanagement Programme eingeführt werden, um die Selbstwirksamkeit der Der DDC zu fördern.   Nächste Schritte/Forschungslücken   - Studien mit größeren Stichproben, die auch Männern einschließen - Qualitative Studien entsprechenden der verschiedenen Type von DDC - Quantitative Langzeitstudien zur Validierung des vorliegenden Konzepts | - Aufgrund der Erwartungen von sich selbst, der Familie und anderen Beschäftigten im Gesundheitswesen fühlen sich DDC dazu verpflichtet, private Pflegaufgaben zu übernehmen - Die meisten DDC sind überzeugt, die einzigen zu sein, die diese Aufgaben übernehmen können. - DDC fühlen sich von ihrer Gesundheitsorganisation nicht ausreichend unterstützt. - DDC fällt es schwer, um Unterstützung zu bitten oder Lösungen zu finden, sind sich aber bewusst, dass Selbstmanagement wichtig ist, um die Situation zu bewältigen. - Einige DDC nehmen Antidepressiva, um durchzuhalten. |
| Fouto & Partington, 2016  Portugal  A, B, C, D | Design  Qualitative Studie  Methode  Halbstrukturierte Interviews  Datenanalyse  Inhaltsanalyse | Gesamtstichprobe  (n=9)  Geschlecht   - Weiblich (n=4) - Männlich (n=5)   Profession   - Pflegefachperson (n=5) - Ärzt:in (n=3) - Röntgenassistent:in (n=1)   Arbeitsort/Funktion  Krankenhaus  Lebenssituation  Keine Angaben  Pflegebeziehung  Keine Angaben | - Kleine Stichprobe - Portugiesische Studie, deren Ergebnisse ins Englische übersetzt wurden – Möglichkeit der Falschinterpretation aufgrund sprachlicher und kultureller Unterschiede | Schlüsselergebnisse  Vier Schlüsselthemen zeigen sich bezogen auf die Wahrnehmungen, Erfahrungen und Bedürfnisse von DDC:   - *Akzeptanz der Pflegerolle* - DDC ist es wichtig, gut zu betreuen, aber auch bei mangelnden körperlichen, emotionalen und finanziellen Fähigkeiten Unterstützung zu erhalten. - *Schwierigkeiten als Pflegende -* Die Zeit der Diagnosestellung ist schwer. DDC möchten die Zukunft gemeinsam planen, fühlen sich aber von der Familie enorm unter Druck gesetzt. - *Belohnungen durch die Ausübung der informellen Pflege* - besonderer Lohn zum Beispiel, wenn sich in dieser Zeit eine vertrauensvolle Beziehung entwickelt. - *Bedürfnisse als Pflegeperson -* Unterstützung bei körperlichen Beschwerden, Ermöglichung sozialer Aktivitäten und Unterstützung bei Entscheidungsfindungen, besonders wenn diese Erfahrungen neu sind.   DDC benennen körperliche, emotionale, soziale, spirituelle sowie Informations- und Kommunikationsbedürfnisse  Nächste Schritte/Forschungslücken  Studien zu Bewältigungsstrategien der festgestellten Bedürfnisse | - Für DDC sind berufliche und private Pflege unterschiedliche Erfahrungen - Die Realität der Rolle von DDC kann für Fachpersonen ein Schock sein - Für DDC ist die private Pflege moralische Verpflichtung, ein Akt des Mitgefühls und/oder eine praktische Annehmlichkeit. - DDC fühlen sich dazu verpflichtet, alles für die gepflegte Person zu tun, was möglich ist. - Alle Berufsgruppen erleben die Pflege ähnlich, besonders die emotionale Einsamkeit und der Wunsch, die Last und die Erfahrungen (Druck durch Familie) mit einer Person zu teilen, die in einer ähnlichen Situation ist - DDC fühlen sich manchmal von der Familie und er Gesellschaft emotional unter Druck gesetzt, da von ihnen erwartet wird, auf alles Antworten parat zu haben und Lösungen zu finden. - Besonders schwierig für DDC ist es, auf Behandlungen zu warten - Sterbebegleitung eines Angehörigen empfinden DDC als Herausforderung und Bereicherung, aber vor allem als Privileg |
| Giles & Williamson, 2015  Australien  A, B, C, D, E, F | Design  Qualitative, deskriptive Studie  Methode  Online-Fragebogen mit drei offenen Fragen und Raum für Erklärungen der anderen Fragen/Antworten  Datenanalyse  Hermeneutische Phänomenologie | Gesamtstichprobe (n=19)  Geschlecht   - Weiblich (n=18) - Männlich (n=1)   Profession  Pflegefachperson  Arbeitsort/Funktion  Keine Angaben  Lebenssituation  Keine Angaben  Pflegebeziehung   - Ehe-/Partner (n=2) - Eltern (n=6) - Schwiegereltern (n=2) - Kinder (n=2) - Geschwister (n=5) - Großeltern (n=1) - Onkel/Tante (n=1) | - Kleine Stichprobe, regional begrenzt - Analyse von Selbstaussagen bezogen auf eine Querschnittstudie, könnte das Antwortverhalten eingeschränkt haben | Schlüsselergebnisse   - DDC benötigen andere Unterstützung als andere pflegende Angehörige - Innerer Konflikt zwischen Verstecken und Offenlegen des eigenen professionellen Hintergrunds - DDC wollen Anerkennung ihres Fachwissens und eine spezialisierte Kommunikation - DDC überwachen und agieren als Fürsprecher - Verschiedene Erwartungshaltungen an sich selbst, von Seiten der Familienmitglieder und professionell Pflegenden - DDC erleben emotionale Belastung aufgrund des Fachwissens, aber auch des Unvermögens, sich von einer der beiden Rollen (Angehörige und Fachperson) vollständig zu lösen, d. h. diese sind untrennbar miteinander verwoben   Nächste Schritte/Forschungslücken   - Forschungen, um festzustellen in welchem Ausmaß DDC von ihren Erfahrungen betroffen sind - Studien aus der Perspektive von Vertretern des Gesundheitswesens | Modell der Erfahrungen von DDC, deren Angehörige mit einer kritischen Krankheit ins Krankenhaus eingeliefert wurden:   - DDC befinden sich einem Doppelrollenkonflikt zwischen Pflegefachperson-Selbst (Nurse-self) versus Familienmitglied-Selbst (Family Member-self) - es sind aber immer beide vorhanden - Ständiger innerer Konflikt, ob der eigene professionelle Hintergrund offengelegt werden sollte oder nicht - DDC haben andere Bedürfnisse als andere pflegende Angehörige, die oft nicht erfüllt werden und dadurch Angst und Belastungserleben auslösen |
| Hansen, 2016  USA  A, B, D, E | Design  Qualitative Studie  Methode  Narrative Interviews, persönlich und per Video  Datenanalyse  Thematische Analyse | Gesamtstichprobe (n=6)  Geschlecht  Weiblich (n=6)  Profession  Pflegefachperson  Arbeitsort/Funktion  Keine Angabe  Lebenssituation   - Selber Haushalt (n=4) - Elternhaushalt (n=2)   Pflegebeziehung   - Ehepartner (n=3) - Eltern (n=2) - Sohn (n=1) | - Narrative Interviews sind nicht reproduzierbar - Möglicher Bias und fehlende Repräsentativität durch freiwillige Teilnahme - Kleine, homogene Stichprobe - Keine männlichen Teilnehmer - Persönliche Erfahrungen der Autorin | Schlüsselergebnisse  *Zentrale Themenkomplexe*:   - *DDCs sind Fürsprecher* - aufgrund ihres Fachwissens und ihrer Kontakte im Gesundheitswesen. Sie sorgen für eine qualitativ hochwertige Pflege für ihre Angehörigen. - *DDC erfahren Lebensveränderungen* - die Profession ermöglicht DDC zu pflegen, aber ihr Leben ändert sich unter diesen Bedingungen ständig und findet Ausdruck in drei Bereichen: finden der Balance zwischen Familie, informeller Pflege und Beruf, damit verbundene Rollenveränderung innerhalb der Familie und Auswirkungen auf die eigene Gesundheit. - *DDC erleben emotionale Belastung* - diese reichen von emotionalem Rückzug und Überwältigung, Dankbarkeit über Wut und Frustration, Schuldgefühle und Trauer über die eigene Rolle   *Stressoren und Mediatoren*:   - Primäre Stressoren: Bedürfnisse der Pflegebedürftigen Angehörigen, I-/ADL Vorgaben, Abhängigkeit von der Pflegeperson, Dauer der Pflege - Sekundäre Stressoren: Konfliktdynamik in der Familie, Arbeitskonflikt, mehrere Rollen, Forderungen der Familie - Mediatoren: soziale Unterstützung, Ressourcen   Nächste Schritte/Forschungslücken   - Fokus auf spezielle Pflegebeziehungen, z.B. nur Eltern oder Ehepartner - Vergleich der Pflege von nahen und entfernten Angehörigen - Fokus auf bestimmte Krankheitsbilder der pflegbedürftigen Personen - Rollenvergleich von Frauen und Männern | - Trotz der immensen Herausforderungen würden die Hälfte der Teilnehmer:innen immer wieder die Pflege von Angehörigen übernehmen - Je länger die private Pflegeaufgabe andauert, desto schwieriger wird es, das eigene Leben im Gleichwicht zu halten und die eigene Gesundheit zu erhalten |
| Häusler et al., 2017  Schweiz  A | Design  Quantitative Querschnittstudie  Methode  Fragebogen mit Teilen aus dem QOPSOQ-Assessment  Definierte Gruppen der informellen Pflege   - Keine (n=849) - Kinder (DDCC) (n=445) - Erwachsene (DDCA) (n=82) - Kinder und Erwachsene (TDC) (n=30)   Datenanalyse  Deskriptiv | Gesamtstichprobe (n=1406)  Geschlecht   - Weiblich (n=1232) - Männlich (n=174)   Profession   - Pflegefachperson und Hebamme (n=854) - Ärzt:in (n=231) - Andere Gesundheitsberufe (n=321)   Arbeitsort/Funktion  Krankenhaus  Lebenssituation  Keine Angaben  Pflegebeziehungen  Keine Angaben | - Keine Darstellung kausale Zusammenhänge aufgrund des Querschnittsdesigns möglich - Rücklaufquote von nur 41% - Die Skala zum Rollenkonflikt fokussiert nur auf Arbeit und Privatleben - Andere Gruppierung der Studiengruppe als in vorherigen Forschungen | Schlüsselergebnisse   - Kaum Unterschiede hinsichtlich Burnout-Risiko und Work-Privacy-Conflict zwischen DDAC und nur professionell Pflegenden feststellbar - Bei TDC ist lediglich das Burnout-Risiko höher als bei den anderen Gruppen   Nächste Schritte/ Forschungslücken   - Größere Studien, die sich auf die Kombination von informellen Betreuungsaufgaben unter Angehörigen der Gesundheitsberufe konzentrieren - Entwicklung eines allgemein anerkannten, gültigen und zuverlässigen Instruments für die informelle Pflege, zur Erhöhung der Vergleichbarkeit von Studien. | - Nur TDC haben ein größeres Burnout-Risiko als alle anderen Beschäftigten im Gesundheitswesen und Schwierigkeiten, die Grenzen wischen beruflicher und privater Pflegeaufgabe zu überwinden - Der Arbeitsbezogene Stress hatte dabei keinen Einfluss auf das Burnout-Risiko der TDC - Zeitliche Beanspruchung durch die Mehrfachrolle und die Unfähigkeit, Arbeits- und Lebensbereiche zu trennen zählen zu den Risikofaktoren für die Gesundheit der DDC |
| Jähnke et al., 2017  Schweiz  D, E, F | Design  Qualitativer Teil einer Mixed-Method-Studie  Methode   - Theoretisches Sampling - Interviews   Datenanalyse  Orientiert an der Grounded Theory | Gesamtstichprobe (n=20)  Geschlecht   - Weiblich (n=17) - Männlich (n=3)   Profession  Pflegefachperson  Arbeitsort/Funktion   - Spitalinterne und -externe Versorgung - Langzeitpflege - Je eine Behörde, Bildungs- und Beratungsinstitution - Überwiegend Führungspersonen   Lebenssituation  Unterschiedlich, keine detaillierten Angaben  Pflegebeziehungen   - Partner:in - Eltern - Geschwister - Kinder | - Es fehlen die Perspektiven von Angehörigen ohne pflegerischen Hintergrund, der involvierten Mitarbeiter:innen sowie der pflegebedürftigen Person - Fokus der Analyse nur auf dem ‚Nutzen‘ der DDC - Starker regionaler Bezug der Erhebung | Schlüsselergebnisse  Strategien bei unerwünschten Ereignissen:   - Vom Beobachten zum Ansprechen (sich als Fachperson positionieren) - Eingreifen und steuern (Präsenz als Kontrollstrategie, Adressieren und Navigieren von MA, Umgang mit Kritik) - DDC erleben Gefühle von Zorn, Hilflosigkeit, Ohnmacht und Ausgeliefert sein, wenn wichtige Informationen, die sie geben, vom Betreuungsteam ignoriert werden   Nächste Schritte/Forschungslücken   - Beobachtungsstudien zu Interaktionen bei unerwünschten Ereignissen - Untersuchungen des Phänomens aus der Perspektiven von bspw. Angehörigen ohne pflegerische Expertise, involvierter Mitarbeiter:innen und pflegebedürftige Personen. - Erfassung, inwieweit DDC selbst zu unerwünschten Ereignissen beitragen könnten | - Zentrales Phänomen bei unerwünschten Ereignissen im KH ist das Dilemma der Positionierung, dies bedeutet die Frage danach, wie positioniere ich mich gegenüber Mitarbeiter:innen im Gesundheitswesen, wenn diese keine adäquate oder sichere Versorgung gewährleisten - greife ich ein bzw. oute ich mich als Pflegefachperson → DDC wissen, wie Pflegefachpersonen über Angehörige denken, die sich einmischen und halten sich deshalb zurück (Sorge bzgl. negativen Sanktionen) - DDC nehmen das unerwünschte Ereignis noch vor dem Betreuungsteam wahr - In lebensbedrohlichen Situationen handeln DDC teils verdeckt, teils offen und geben Handlungsanweisungen - Präsenz zeigen beschleunigt die Prozesse - DDC nehmen ihre Erfahrungen mit in ihren Arbeitsalltag und werden zu Fürsprechern für Angehörige ihrer Patienten - DDC scheinen trotz oder wegen ihrem professionellen Hintergrund eine vulnerable Gruppe zu sein - schwierig den Grat zwischen zu viel und zu wenig Information zur Patientensituation zu finden |
| Jones, 2020  USA  D, E, F | Design  Qualitative, phänomenologische Studie  Methode  Interviews und Feldnotizen  Datenanalyse  Thematische Analyse, Member-Checking | *Gesamtstichprobe* (n=25)  *Geschlecht*   - Weiblich (n=22) - Männlich (n=3)   Profession  Pflegefachperson  Arbeitsort/Funktion  Aktuelles Beschäftigungsverhältnis, egal welcher Arbeitsort  Lebenssituation  Keine Angaben  Pflegebeziehungen   - Eltern (n=10) - Ehepartner (n=9) - Großeltern (n=2) - Schwiegermutter/-vater (n=2) - Onkel/Tante (n=1) - Geschwister (n=1) | - Gezielte Stichprobe - Nur Pflegefachpersonen und keine andere Gesundheitsberufe einbezogen - Erhebung in nur einem Bundesstatt, deshalb limitierte Übertragbarkeit (kulturelle Unterschiede etc.) | Schlüsselergebnisse  Kernaussage: Beide Rollen sind untrennbar miteinander verbunden  Fünf identifizierte Haupthemen:   - *Verschwimmende Grenzen* *(‚blurring boundaries‘)* - *Offenbarung meiner Identität* *(‚revealing my identity‘)* - *Insiderwissen haben* *(‚having insider knowledge‘)* - *Erwartungen managen* *(‚managing exspectations‘)* - *aus den Erfahrungen lernen* *(‚learning from the experience‘)*   Nächste Schritte/Forschungslücken  Untersuchung der Erfahrungen/Erleben von anderen Mitarbeitern im Gesundheitswesen in ähnlichen Situationen | - Die zwei Rollen der DDC sind untrennbar miteinander verbunden - DDC ringen mit der Entscheidung, ob sie sich als vom Fach offenbaren sollen oder nicht - DDC lernen von den eigenen Erfahrungen und passen daraufhin ihre Berufspraxis an - Nur wenn DDC dem Pflegepersonal vertrauen, können sie entspannen und in die Rolle der Familienangehörigen schlüpfen |
| Klages et al., 2020a  Australien  D, F  Artikel 1 zur Dissertation von Klages, D. | Design   - Qualitative Studie - Sozial-konstruktivistisches Forschungsparadigma   Methode   - Interviews nach der Storytelling- Methode und Feldnotizen - Via Messenger oder in persona   Datenanalyse  Reflexive thematische Analyse | Gesamtstichprobe (n=13)  Geschlecht  Weiblich (n=13)  Professionen   - Psychiatrische Pflegefachperson (n=4) - Allgemeine Pflegefachperson (n= 5) - Medizinforscherin (n=1) - Ergotherapeutin (n=1) - Sozialarbeiterin (n=1) - Ärztin (n=1)   Arbeitsort/Funktion  Keine Angaben  Lebenssituation  Keine Angaben  Pflegebeziehung  Erwachsene Kinder mit Schizophrenie | - Fehlende kulturelle Vielfalt - Kleine Stichprobe - Umfang und Design der Studie als Einflussfaktor auf die Teilnahmebereitschaft | Schlüsselergebnisse  *Übergeordnetes Thema:*  Bemuttern im Kontext der Unsicherheit  *Haupt- und Subthemen:*  1. gestörte Mutterrolle   - Leben mit Angst und Not - Allein durch das Unbekannte navigieren - Zweideutige/zwiespältige Hoffnung wecken (*Creating ambiguous hope)*   2. Rekonfigurierte Mutterrolle   - Kämpfen für ihr Recht, gehört zu werden - Nutzung ihrer Stärken als Fachpersonen - Umgestaltung der mütterlichen Praxis   3. Entschlossene Mutterrolle   - Sinn im gestörten Leben finden. - Reflexion der Tatsache, einen Fuß in beiden Welten zu haben - Die Kosten der Pflege tragen   Nächste Schritte/Forschungslücken  Forschungen mit Gesundheitsfachkräften, die Mütter bei der Betreuung ihrer Kinder im Rahmen einer Zusammenarbeit mit psychosozialen Diensten unterstützen | - DDC entwickeln trotz der Herausforderungen eine unklare Hoffnung für die Zukunft ihrer Kinder, wobei die Ängste aber bestehen bleiben - DDC akzeptieren stillschweigend ihre aktuellen Lebensumstände und setzten ihre Stärken und verfügbare Ressourcen ihrer psychischen Gesundheit ein - Gerade DDC mit psychiatrischen Fachkenntnissen fühlen sich in eine Mutterrolle gedrängt, die sie nicht wollen, dadurch dass sie ihre Fürsorgerolle an ‘Experten‘ abgeben sollen - DDC wollen mehr in die Betreuung mit einbezogen werden - Die Übernahme der Funktion als ‚Case Managerin‘ ist zwar von Vorteil für die Kinder, aber da beide Rollen verschwimmen, wird nicht mehr auf die Bedarfe der DDC geachtet |
| Klages et al., 2020b  Australien  D  Artikel 2 zur Dissertation von Klages, D. | Design   - Qualitative Studie - Sozial-konstruktivistisches Forschungsparadigma   Methode   - Interviews nach der Storytelling- Methode und Feldnotizen - Via Messenger oder in persona   Datenanalyse  Reflexive thematische Analyse | Gesamtstichprobe (n=13)  Geschlecht  Weiblich (n=13)  Professionen   - Psychiatrische Pflegefachperson (n=4) - Allgemeine Pflegefachperson (n= 5) - Medizinforscherin (n=1) - Ergotherapeutin (n=1) - Sozialarbeiterin (n=1) - Ärztin (n=1)   Arbeitsort/Funktion  Keine Angaben  Lebenssituation  Teils zuhause, teils woanders  Pflegebeziehung  Erwachsene Kinder mit Schizophrenie | - Beschränkung auf Professionen im Gesundheitswesen und Mütter - Beschränkung auf Erwachsene Kinder - Beschränkung auf die Erkrankung Schizophrenie | Schlüsselergebnisse  Es gibt vier Typen von Müttern, orientiert am Grad ihrer Involviertheit in der Pflege:   - *ständige Betreuerin (‚constant carer‘)* - hohes Maß an Pflege, Kinder leben zuhause - *koordinierende Betreuerin (‚coordinator carer‘)* - moderates Maß an Pflege, Kinder leben zuhause oder woanders - *wachsame Beobachterin (‚watchful bystander‘)* - geringeres Maß an Pflege, Kinder leben zuhause oder woanders - *die Lebensberaterin (‚life coach carer‘)* - minimales Maß an Pflege, Kinder leben woanders   Zu den Aspekten der Pflege, die von allen Typen in unterschiedlicher Intensität geleistet werden, gehören: Überwachung, Kommunikation, Unterbringung, Finanzen/Rechtliches, Fachwissen und Bemutterung (S. 3)  Nächste Schritte/Forschungslücken   - Einschluss anderer Familienmitglieder als DDC - ggf. anderes Rollenverständnis - Einschluss von Männern im Gesundheitswesen - Rollenverständnis von Vätern - Betreuung von Kindern mit anderen psychiatrischen Erkrankungen | - DDC sind wichtig als Informanten, da sie eine lebenslange Verbindung zu ihren Kindern haben und detailliertes Wissen über die Krankengeschichte oder sozialer Aspekte haben. Deshalb erkennen sie Warnzeichen frühzeitig, was ihre Funktion vergleichbar mit einer Case Managerin macht. - Die DDC sind zwar lebenslang einbezogen, ihre Expertise wird bisher aber nicht wahrgenommen und wertschätzt - DDC erleben im Verlauf Veränderungen ihrer eigenen Identität sowie ihrer Rolle. |
| Klages et al., 2020c  Australien  A  Artikel 3 zur Dissertation von Klages, D. | Design   - Qualitative Studie - Sozial-konstruktivistisches Forschungsparadigma   Methode   - Interviews nach der Storytelling- Methode (feministischer Erzählansatz) und Feldnotizen - Via Messenger oder in persona   Datenanalyse  Reflexive thematische Analyse | Gesamtstichprobe (n=13)  Geschlecht  Weiblich (n=13)  Profession   - Psychiatrische Pflegefachperson (n=4) - Allgemeine Pflegefachperson (n= 5) - Medizinforscherin (n=1) - Ergotherapeutin (n=1) - Sozialarbeiterin (n=1) - Ärztin (n=1)   Arbeitsort/Funktion Keine Angaben  Lebenssituation  Keine Angaben  Pflegebeziehung  Erwachsene Kinder mit Schizophrenie | Keine Angaben | Schlüsselergebnisse  Themen im Zusammenhang mit posttraumatischen Entwicklungen (Vermischung von Fach- und persönlichem Wissen) (S. 4f):   - *uns waren die Hände gebunden (‚our hands were tied‘)* - die DDC wissen von Anfang, wenn etwas nicht stimmt, da sie die Warnzeichen bemerken, erhalten aber keine Hilfe oder Unterstützung - *akzeptieren und anpassen (‚accepting and adapting‘)* - DDC lernen, die veränderten Lebensumstände zu akzeptieren und sich daran anpassen - *ich könnte den Unterschied bewirken (‚I could make the difference‘)* - DDC stellen fest, dass das eigene erweiterte Wissen genutzt werden kann, um anderen zu helfen und zur Verbesserung der Gesamtsituation beizutragen   Nächste Schritte/Forschungslücken  Notwendigkeit, das Wissen über Fachpersonal im Gesundheitssektor, die Mütter von erwachsenen Kindern mit Schizophrenie sind, weiter zu entwickeln und zu erforschen. | - DDC können, wenn sie trotz der traumatischen Ereignisse positive Veränderungen erleben, in der Folge den Drang verspüren, andere Personen in ähnlicher Situation zu unterstützen. Durch das posttraumatische Trauma erleben die DDCs sowohl Stress als auch ein Anwachsen der persönlichen Stärke. - Die Vermischung beider Wissengrundlagen (fachliches und das durch die Mutterschaft von an Schizophrenie erkrankter Kinder erlangtes Wissen) stärkt beide Wissensarten. |
| Mills & Aubeeluck, 2006  England  B, C, D, E, F | Design  Qualitative Studie  Methode  Halbstrukturierte Interviews  Datenanalyse  Interpretative phänomenologische Analyse | Gesamtstichprobe (n=5)  Geschlecht  Weiblich (n=5)  Profession  Pflegefachfrau  Arbeitsort/Funktion   - Akutkrankenhaus - Führungskräfte   Lebenssituation  Keine Angaben  Pflegebeziehung  Angehörige mit einer diagnostizierten lebensbedrohlichen Erkrankung | - Kleine Stichprobe - Alle Teilnehmerinnen waren Pflegende in einem Akutkrankenhaus - Alle waren Führungspersonen - Beeinflussung der Analyse aufgrund der therapeutischen Beziehungen zwischen der Forscherin und den Teilnehmerinnen | Schlüsselergebnisse  Vier übergeordnete Themen wurden ermittelt   - *Lebensqualität* - in der Regel negativ beeinflusst - *Persönliche und professionelle Grenzen* - Wissen früher über Konsequenzen Bescheid, sollen informieren, können die Rollen nicht trennen - *Entmachtung/Entmündigung* - machtlos gegenüber prof. Pflegenden, eigene Bedürfnisse werden nicht berücksichtigt - *Positive Aspekte der Rolle* - berufliche Fähigkeiten kommen dem Angehörigen zu Gute, reflektierter Blick auf die eigene Arbeit   Nächste Schritte/Forschungslücken   - Quantitative Forschungen, die unterschiedliche Gruppen DDC betrachten und vergleichen - Längsschnittstudien zur Erfassung der längerfristigen Auswirkungen | - DDC schauen voraus, was zur Isolation führt, da sie ihre Gefühle nicht ausdrücken können, nicht pessimistisch wirken und ihren Angehörigen weiter unterstützen wollen - DDC fühle sich überfordert, wenn von ihnen Fachwissen abverlangt wird, welches sie nicht haben - DDC trauen sich nicht Unzufriedenheit mit der Pflege durch Fachpersonal zu thematisieren, um nicht als kritisch wahrgenommen zu werden - Die Erfahrungen der Pflege in beiden Feldern beeinflussen das eigene Handeln im jeweils anderen Bereich |
| Quinney et al., 2018a  Australien  D, E  Dieselbe Datengrundlage wie Quinney et al., 2018b | Design  Qualitative Studie  Methode  Unstrukturierte Interviews  Definierte Gruppe der informellen Pflege   - Pflege von Familienmitgliedern mit einer chronischen Erkrankung, mindestens 12 Monate in den letzten fünf Jahren - mindestens 5 Jahre Berufserfahrung   Datenanalyse  Hermeneutische Analyse | Gesamtstichprobe (n=15)  Geschlecht   - Weiblich (n=13) - Männlich (n=2)     Profession  Pflegefachperson  Arbeitsort/Funktion  Keine Angabe  Lebenssituation  Keine Angabe  Pflegebeziehung   - Kinder (n=7) - Eltern (n=5) - Ehemann (n=2) - Enkelkind (n=1) | - Beschränkung auf eine Region Australiens - Nur zwei männliche Teilnehmer | Schlüsselergebnisse   - Die Angst um das kranke Familienmitglied macht DDC zu: a) *Vermittlern* zwischen Beschäftigten im Gesundheitswesen und krankem Familienmitglied b) *Navigatoren* durch das Gesundheitssystem, um Fachpflege zu erhalten c) *Beschützern* aus Angst, z.B. beim Krankenhausaufenthalt (Fürsprecher, Überwacher)   Nächste Schritte/Forschungslücken   - Identifizierung der Erleichterungen und Hindernisse für eine erfolgreiche Zusammenarbeit zwischen DDC und Angehörigen der Gesundheitsberufe - Erfassung der Verbreitung des Phänomens der Betreuung von chronisch Erkrankten durch DDC | - Fachwissen verursacht Ängste vor bekannten Gefahren und Unbekanntem - die Rollen der Vermittler, Navigatoren und Beschützer sind miteinander verbunden, voneinander abhängig, aber nicht hierarchisch und DDC wechseln zwischen ihnen je nach den Bedürfnissen ihrer Angehörigen - DDC agieren ähnlich wie Case Manager:innen |
| Quinney et al., 2018b  Australien  A, B, D, E, F  Dieselbe Datengrundlage wie Quinney et al., 2018a | Design  Qualitative Studie  Methode  Unstrukturierte Interviews  Definierte Gruppe der informellen Pflege   - Pflege von Familienmitgliedern mit einer chronischen Erkrankung, mindestens 12 Monate in den letzten fünf Jahren - Mindestens 5 Jahre Berufserfahrung   Datenanalyse  Phänomenologisch-hermeneutische Analyse | Gesamtstichprobe (n=15)  Geschlecht   - Weiblich (n=13) - Männlich (n=2)   Profession  Pflegefachperson  Arbeitsort/Funktion  Keine Angabe  Lebenssituation  Keine Angabe  Pflegebeziehung   - Kinder (n=7) - Eltern (n=5) - Ehemann (n=2) - Enkelkind (n=1) | - Beschränkung auf eine Region Australiens - Nur zwei männliche Teilnehmer | Schlüsselergebnisse  Die Erfahrungen von DDC beziehen sich auf drei thematische ‚Welten‘ (in Anlehnung an Heidegger): die Welten des *persönlichen Seins*, des *beruflichen Seins* und des *praktischen Seins*.  Hier im Fokus die Welt des persönlichen Daseins, die sich bildet aus:   - *geteilte Erfahrungen* - ständiger Begleiter, Verlust und Veränderung - *Pflegeerfahrung* - Pflegehindernisse, Wissen und Tun - *belastende Erfahrungen* - immer wachsam sein, Schuldgefühle, 'Fließband' der chronischen Krankheiten   Nächste Schritte/Forschungslücken  Aufbau einer Familienberatung, die bei der Aufnahme psychologische, soziale und praktische Unterstützung gibt | - DDC möchten nicht nur einfach Familienmitglied sein, sondern empfinden durch ihre Beziehung zu ihrem Angehörigen und in ihrer Eigenschaft als Pflegfachperson die Begleitung ihres kranken Familienmitglieds als einen wichtigen Teil ihres Lebens und ihrer Art des *‚Seins‘.* - DDC beschreiben die Betreuung als langwierig und dauerhaft - Durch die Fortlaufende Aufgabe ist es für DDC wichtig, eine positive Beziehung zum Personal aufzubauen und zu halten, ansonsten erleben sie Spannungen und Frustration - DDC erleben Positives, wie Zusammenhalt, aber auch *emotionale* *Turbulenzen*, wie Schuldgefühle, Furcht und Angst |
| Salmond, 2011  USA  A, B, D, E, F | Design  Qualitative Studie  Methode   - Offene, leicht strukturierte Interviews - Theoretical Sampling   Datenanalyse  Angelehnt an Grounded Theory | Gesamtstichprobe (n=22)  Geschlecht  Weiblich (n=22)  Profession  Pflegefachperson  Arbeitsort/Funktion   - Stationspersonal (n=12) - Leiterinnen (n=4) - Ausbilderinnen (n=6)   Lebenssituation  Keine Angaben  Pflegebeziehung   - Erwachsende Verwandte, die in den letzten 2 Jahren aufgrund einer lebensbedrohlichen, kritischen Erkrankung oder Ereignisses auf einer Intensivstation lagen - Kinder - Enkel - Geschwister - Eltern - Ehemann - Schwiegereltern - Onkel/Tante | - Keine männlichen Teilnehmer - Wenig bis keine Konflikte innerhalb der Familien | Schlüsselergebnisse   - Konzeptuelles Modell: Die Reaktion von pflegenden Familienmitgliedern auf die kritische Krankheit eines geliebten Menschen. - Kernkategorie: Professionelle und private Rolle sind miteinander verwoben, doch die meisten sind mehr die Pflegefachperson - DDC sind dabei sowie Fürsprecher - DDC wünschen detaillierte Informationen Vorteile gab es, wenn Angehörige im eigenen KH stationär waren, so wurden sie mit einbezogen   Nächste Schritte/Forschungslücken   - Die gleiche Forschung mit Fokus auf männliche DDC - Forschung zu den Komponenten des Modells für ein tieferes Verständnis - Empirische Überprüfung des Modells - Ergänzende qualitative Untersuchung der Wahrnehmung von Intensivpflegefachpersonen bei der Pflege von Patienten, deren Familienmitglied auch Pflegefachperson ist | - Um die Kontrolle zu behalten verbergen DDC ihre Emotionen, wie Ängste, nicht nur vor den Pflegebedürftigen, sondern auch vor anderen Familienmitgliedern und dem Personal - Das *Dabeisein* ist elementar für die Überwachung und Beurteilung der Situation, verursacht aber auch Stress - Die Erfahrung ins Stationsteam ‚eingelassen‘ zu werden (‚being let in‘) schafft Vertrauen und Zuversicht |
| Santerre-Theil et al., 2022  Kanada  A, B, D | Design   - Qualitative Studie - Exploratives, deskriptives Design   Methode  Halbstrukturierte Interviews  Definierte Gruppen der informellen Pflege  DDC, die Angehörige mit einer onkologischen Erkrankung, welche eine Behandlung (Chemo, Bestrahlung, Operation) erhielten, in den letzten 10 Jahren betreut haben  Datenanalyse  Thematische Analyse | Gesamtstichprobe (n=12)  Geschlecht   - Weiblich (n=11) - Männlich (n=1)   Profession   - Pflegefachfrau (n=7) - Ärzt:in (n=5) - mindestens 1 Jahr Berufserfahrung   Arbeitsort/Funktion  Keine Angaben  Lebenssituation  Keine Angaben  Pflegebeziehung   - Partner (n=3) - erwachsene Kinder (n=4) - Geschwister (n=1) - Eltern (n=1) - Schwiegereltern (n=2) - Großvater (n=1) | - Nur ein männlicher Teilnehmer – Fokus auf die weibliche Perspektive - Keine Rücksprache mit den Interviewpartnern, um Bedeutung von Wortlaut zu klären oder zu vertiefen - Beschränkt auf Ärzte und Pflegende und keine anderen Professionen in der Onkologischen Pflege | Schlüsselergebnisse  *Vorteile des Fachwissens*   - Informationen erhalten und verstehen (eine Sprache sprechen) und diese den Angehörigen und anderen Familienangehörigen erklären können - Sich im Gesundheitssystem auskennen und Wissen, an wen sie sich wenden können ist hilfreich   *Besondere Herausforderungen*   - Fühlen sich von der Familie, Professionellen und sich selbst unter Druck gesetzt, ihre Rolle wahrzunehmen. - Fachwissen führt dazu, dass Verschlechterungen und Versorgungslücken frühzeitig erkannt und die Verantwortung übernommen werden. - Gefühl haben, nicht alles tun zu können. - Konfrontation mit den Lücken im Gesundheitssystem. - Keine Erholung von der Pflege haben. - Liegen Angehörige im eigenen Krankenhaus und erfahren DDC während der Arbeitszeit von gesundheitlichen Problemen, ist es schwierig weiterzuarbeiten   *Veränderungen der eigenen Arbeitsweise in Folge von DDC*   - Besseres Verständnis für Erkrankte und ihre Familien, in der Folge zugewandterer und emphatischerer Umgang sowie stärkerer Einbezug aller Beteiligten in die Pflege - veränderte Kommunikation - Lernen von Fachpersonen im onkologischen Bereich für das eigene Tun   *Palliativ Pflege*  Wunsch nach frühzeitigem Zugang zu und Unterstützung bei der palliativen Pflege  Nächste Schritte/Forschungslücken   - Erfassung von spezialisierten Unterstützungsbedarfen in unterschiedlichen Kontexten - Erfassung der Erfahrungen von DDC im Krankheitsverlauf, einschließlich Überlebensphase und über den Tod hinaus - Entwicklung, Testung und Umsetzung von personenzentrierten unterstützenden Interventionen | - Im eigenen Haus schwierig, wenn während der Arbeitszeit von gesundheitlichen Problemen erfahren, dann schwierig weiterzuarbeiten - Der professionelle Hintergrund hindert sie am Trauern, da sie glauben, für alle stark sein zu müssen. |
| St-Amant et al., 2014  Kanada  A, B, D, E, F  Dieselbe Datengrundlage wie Ward-Griffin et al., 2015 | Design   - Qualitative Studie - Phase II einer sequenziellen Mixed-Method-Studie   Methode   - Telefoninterviews an zwei Zeitpunkten - Fokusgruppen (n=2)   Definierte Gruppen der informellen Pflege   - ‚damit es gelingt‘ (n=4/7) - ‚arbeiten um zurecht zu kommen‘ (n=11/11) - ‚am Rande leben‘ (n=17/14)   (vgl. Ward-Griffin et al., 2005)  Datenanalyse Komperative Datenanalyse mittels konstruktivistischer Grounded Theory | Gesamtstichprobe  T0 (n=50), **T1 (n=32)**, Fokusgruppen (n=11)  Geschlecht  Weiblich (n=32)  Profession  Pflegefachperson  Arbeitsort/Funktion  Keine Angaben  Lebenssituation  Keine Angaben  Pflegebeziehung  Keine Angaben | Keine Angaben | Schlüsselergebnisse   - Übergeordnete Strategien professionalisierter informeller Pflege: Grenzen setzen (z.B. für Art der Pflege und Angemessenheit der Aufgabe) und Verbindungen zu Akteuren im System herstellen - Voneinander abhängige untergeordnete Strategien: 1. *Bewerten (assassing)* - objektive Beurteilung der Pflegesituation und Entscheidungsfindung 2. *Beraten/Unterstützen (advising)* - der Pflegeperson und der Familie 3. *Fürsprechen (advocating)* - führt zu Konfrontation, aber auch Zusammenarbeit mit den Leistungserbringer:innen 4. *Zusammenarbeiten (collaborating)* - um eine angemessene, nahtlose und qualitativ hochwertige Pflege zu gewährleisten 5. Beraten (consulting) - der Familie 6. Koordinieren (coordinating)   Nächste Schritte/Forschungslücken   - Auswirkungen von DDC auf die familiären Beziehungen jeweils aus der Perspektiven von DDC und Familienangehörigen - Verständnis der Professionalisierung der Pflege erlangen durch Untersuchungen, wie Personen anderer Berufe ihre Kompetenzen im Familienkontext einsetzen | - DDC wenden dieselben Strategien in der formellen und informellen Pflege an, bei letzterer jedoch ohne rechtliche Regelungen und Absicherung. - Viele DDC haben das Gefühl, endlos zu arbeiten und dass Erholungsphasen fehlen - der professionelle Hintergrund und die dadurch objektive Bewertung der Situation hilft Grenzen zu setzen, bzgl. der Beratung von Familienmitgliedern, sowie Entscheidungen zu treffen, welche Infos weitergegeben werden und welche Informationen sie selbst benötigen - manche DDC vermeiden es, sich als Pflegefachperson zu erkennen zu geben und können sich so nicht mit anderen Berufsangehörigen beraten - DDC beklagen, dass ihnen zuhause nicht die Ressourcen zu Verfügung stehen, diese auf der Arbeit haben und fühlen sich unwohl, wenn sie sich für die Aufgabe nicht qualifiziert fühlen - DDC erfahren keine Anerkennung ihrer Pflegeübernahme außerhalb der Familie |
| Ward-Griffin, 2004  Kanada  E | Design   - Explorative, qualitative Studie - Sozialistisch-feministische Perspektive   Methode   - Narrative Interviews - Feldnotizen 2 Follow-up Fokusgruppen (n=13)   Definierte Gruppen der informellen Pflege  Pflegeübernahme von mindestens eine Stunde pro Woche  Datenanalyse  Nach den Empfehlungen von Lofland und Lofland 1995 | Gesamtstichprobe (n=15)  Geschlecht  Weiblich (n=15)  Profession  Pflegefachfrau (83%)  Arbeitsort/Funktion   - Kommunaler Gesundheitsdienste - ambulante Pflege (66%)   Lebenssituation  Überwiegend getrennte Haushalte  Pflegebeziehung   - Eltern (66%) - Schwiegereltern (13%) - Großeltern (7%) - Schwester (7%) - Ehepartner (7%)   1/3 betreute zwei oder mehr ältere Angehörige | Keine Angaben | Schlüsselergebnisse   - DDC vereinen zwei Dimensionen der Pflege:  *caring about* (Sorge um) und *caring for* (Sorge für), diese sind untrennbar miteinander verwoben - Beide Formen der Sorge kommen in beiden Bereichen vor, in unterschiedlicher Ausprägung - Theorieentwicklung - Notwendig, Maßnahmen durch Programme zu überprüfen sowie Führungskräfte zu sensibilisieren, um den Stress für DDC zu verringern und gesundheitsfördernde Maßnahmen zu unterstützen   Nächste Schritte/Forschungslücken   - Erfassung positiver oder negativer Auswirkungen auf DDC durch Art und Umfang der Beschäftigung - Verwendung eines feministischen Rahmens in der Forschung, um die kritische Reflexion unter den DDC zu fördern - Vergleich der Erfahrungen von Frauen und Männern | - Pflegende glauben, dass man nicht gleichzeitig *caring about* (Sorge um) und *caring for* (Sorge für) betreiben kann - Die emotionale Bindung zu den Angehörigen beeinträchtig das klinische Urteilsvermögen - DDC treffen manchmal widersprüchliche Aussagen - Geschlechtsspezifische Ideologien nehmen maßgeblich Einfluss auf das Leben von weiblichen DDC |
| Ward-Griffin et al., 2015Kanada B, D, E  Dieselbe Datengrundlage wie St.-Amant et al., 2014 | Design  Qualitative Studie (Phase II einer sequenziellen Mixed-Method Studie)  Methode   - Halbstrukturierte Telefon-Interviews - Abschließende Fokusgruppen   Definierte Gruppen der informellen Pflege   - making it work - working to manage - living on the edge   Datenanalyse   - Konstant vergleichende, konstruktivistische Grounded Theory - Je eine Fallvignette pro Gruppe/Typ | Gesamtstichprobe (n=50)   - Interviews T1 (n=50) - **Interviews T2 (n=32)** - Fokusgruppen (n=11)   Geschlecht   - Weiblich (n=16) - Männlich (n=16)   Profession  Keine Angaben  Arbeitsort/Funktion  Keine Angaben  Lebenssituation  Keine Angaben  Pflegebeziehung  Keine Angaben | Keine Angaben | Schlüsselergebnisse   - Theorie des Aushandelns beruflicher und familiärer Grenzen in der Pflege - Die Aushandlungsprozesse sind individuell und orientieren sich im Verlauf an Erwartungen und Ressourcen - Die Schnittmenge im Aushandlungsprozess - DDC setzen Grenzen und stellen Verbindungen her, was den Grad des Aufweichens der Grenzen zwischen beruflicher und privater Pflege bestimmt - DDC haben sechs Strategien, um mit den an sie gestellten Anforderungen umzugehen: Einschätzung der Situation, Beraten, Fürsprechen, Zusammenarbeit (mit anderen im Gesundheitswesen), Koordination und Organisation sowie selbst Beratung einholen - Diejenigen, die es schaffen, sind am wenigsten gesundheitlich belastet…   1. wenn die soziale Bedeutung des Verschwimmens der Grenzen nicht beachtet wird, kann das gesundheitliche Folgen für DDC haben  2. Fehlende Unterstützung von DDC kann weitreichende Folgen für die Humanressourcen im Gesundheitsbereich haben und somit ein Risiko der Pflegebedürftigen in beiden Bereichen haben  Nächste Schritte/ Forschungslücken  Welchen Nutzen haben flexible Arbeitsbedingungen tatsächlich | - Die Rollen/Erfahrungen der DDC verändern sich im Verlauf - Im Beruf setzen sie Grenzen, im privaten Bereich stellen sie Verbindungen her - Die Schnittstelle ist durch die dialektischen Prozesse *Professionalisierung der familiären Pflege* und *Streben nach Balance* geprägt - Die Suche nach Balance wird bestimmt durch Anforderungen und Selbstzweifel, anderen gerecht zu werden, eigenes Wissen. Je nachdem wie gut das funktioniert, sind sie einem der drei Typen zuzuordnen. Im Verlauf kommt es immer wieder zu Verschiebungen - Das Verschwimmen der Grenzen wird bestimmt durch veränderte Pflegebedürfnisse, Erwartungen und Ressourcen - Frauen befinden sich ehr an der Belastungsgrenze, besonders im Verlauf, als Männer |
| Ward-Griffin et al., 2005  Kanada  D  Grundlage für Ward-Griffin et al., 2011 | Design   - Explorativer qualitativer Ansatz - Feministische, narrative Studie   Methode   - Fokussierte (Tiefen)Interviews - Feldnotizen Demografische Daten (n=37) - Drei Follow-up Fokusgruppen (n=21)   Datenanalyse  Nach den Empfehlungen von Lofland und Lofland 1995 | Gesamtstichprobe (n=37)  Geschlecht  Weiblich (n=37)  Profession   - Pflegefachfrau (n=15) - Ärztin (n=6) - Physiotherapeutin (n=7) - Sozialarbeiterin (n=9)   Arbeitsort/Funktion  Verschiedene kommunale Gesundheitsdienste  Lebenssituation  Keine Angaben  Pflegebeziehung   - Schwieger-/Eltern - Großeltern - Geschwister - Freunde - Tanten - Ehepartner:innen   ca. 1/3 betreuten 2 oder mehr ältere Angehörige | - Es wurden nur gutverdienende Beschäftigte im Gesundheitswesen befragt - Durch Querschnittdesign nur Abbildung der aktuellen Situation möglich | Schlüsselergebnisse  DDC enthält drei wesentliche Komponenten: 1. *Erwartungen an die familiäre Pflege* - DDC glauben, keine andere Wahl zu haben und fühlen sich isoliert 2. *Unterstützungsgrad* - besseres Gelingen von DDC, wenn mehr Zeit, finanzielle Mittel und Ressourcen vorhanden sind 3. *Verhandlungsstrategien* - die Grenzen zwischen professioneller und familiärer Pflege verschwimmen dabei  Typen von DDC, basierend auf den Erfahrungen:  1. *Es schaffen* (‚making it work‘) 2. *Daran arbeiten, zurechtzukommen* (‚working to manage‘) 3. *Am Rande des Abgrunds leben* (‚living on the edge‘)  Nächste Schritte/Forschungslücken   - Auswirkungen unterschiedlicher Einkommen auf DDC, besonders in der Langzeitpflege (soziale Ungleichheit) - Interviews mit mehr Personen, um das breite Spektrum besser abbilden zu können - Große systematische Studie zur Erfassung der Verteilung der Typen in den verschiedenen Berufsgruppen - Eine Zusammenarbeit von Entscheidungsträgern aus Politik, Öffentlichkeit, Fachkreisen und Wissenschaft zur Entwicklung von Strategien ist erforderlich | - Von DDCs wird erwartet, dass sie im privaten Rahmen unbezahlte professionelle Hilfe leisten (müssen), was sie als ungerecht empfinden - DDC ein dynamischer Prozess ist und DDCs wechseln im Betreuungsverlauf zwischen den drei Typen, je nach Erwartungen der Familie, der erhaltenen Unterstützung und Aushandlungsstrategien. |
| Ward-Griffin et al., 2011  Kanada  A, B, C, D, E  Sekundäranalyse der Daten von Ward-Griffin et al., 2005 und Ward-Griffin et al, 2009 | Design  Qualitative Sekundäranalyse  Methode  Interviews (n=25)  Datenanalyse  Kritischer, feministischer Ansatz nach Lofland et al. 2006 | Gesamtstichprobe (n=20)  Geschlecht  Weiblich (n=20)  Profession  Pflegefachfrau  Arbeitsort/Funktion  Keine Angaben  Lebenssituation  Keine Angaben zu Wohnort  Der Gruppe der ‚Living on the edge‘ zugehörig  Pflegebeziehung   - Ältere Eltern - 56% betreuten zwei oder mehr ältere Angehörige | Keine Angaben | Schlüsselergebnisse   - Kontext von Mitleidsermüdung: Erwartungen und Ressourcen von DDC - Unterstützung durch Familie, Kollegen und Professionelle Dienste - Definierende Merkmale der Mitleidsermüdung: Emotionen, Zuneigung, Identifikation mit dem Pflegebedürftigen, Fachwissen, intensive und lang andauernde Pflege - Folgen von Mitleidsermüdung im Rahmen von DDC: Krankheitssymptome sowie körperliche und geistige Erschöpfung - DDC werden aufgrund ihrer Profession ‚genötigt‘ sich um sich selbst zu kümmern oder den Zustand zu akzeptieren (‚dem Opfer die Schuld geben‘) - Keine Wahl haben, Unzulänglichkeit und Ohnmacht, Pflegeverantwortung haben   Nächste Schritte/Forschungslücken   - Verbindung zwischen beruflicher und familiärerer Pflege sowie deren Auswirkungen erforschen - Anbieter, Führungskräfte und Politiker sollten sich mit sozialpolitischen Faktoren die DDC betreffend auseinandersetzen, um den Auswirkungen der Mitleidsermüdung entgegenzuwirken | - Durch die berufliche und private Pflegeaufgabe als Töchter können sie der Pflegerolle nicht entkommen - dies sollte berücksichtigt werden - Es herrscht eine Diskrepanz zwischen den Erwartungen an DDC und den ihnen in der Häuslichkeit zu Verfügung stehenden Ressourcen - Die Genderproblematik spielt immer wieder mit rein und führt zu Ungleichheiten auch im Rahmen der erteilten Unterstützung |
| Wilson & Ardoin, 2013  USA  A, D, E | Design  Fallstudie  Methode  Persönliche Berichte  Datenanalyse  Analyse und Reflexion nach einem ganzheitlichen Ansatz | Gesamtstichprobe (n=2)  Geschlecht  Weiblich (n=2)  Profession  Sowohl Pflegefachfrau als auch Pflegewissenschaftlerin  Arbeitsort/Funktion  Keine Angaben  Lebenssituation  Getrennte Haushalte  Pflegebeziehung   - Vater (n=1) - Schwiegermutter (n=1) | Keine Angaben | Schlüsselergebnisse   - Beide Autorinnen beschreiben ihre persönlichen Erfahrungen der Pflege eines Angehörigen, reflektieren gemeinsam die jeweiligen Situationen und beschreiben ihre Coping-Strategien - Patientenverfügungen waren hilfreich bei der Entscheidungsfindung - Die Zusammenarbeit der einen Familie mit dem Arzt war gut, da der Vater selbst Arzt war - Schlaflosigkeit führte zur Erschöpfung   Nächste Schritte/Forschungslücken  Keine Angaben | - Beide Autorinnen erlebten antizipatorische Trauer mit einerseits gleichen Auswirkungen, wie Schlafstörungen, Müdigkeit, Sorgen, Traurigkeit, Hilflosigkeit und Überforderung, aber unterschiedlichen: eine Autorin unterdrückte die Trauer, um für andere da zu sein und lies keine fremde Unterstützung zu. Die andere zeigte ihre Gefühle offen und war dadurch Tochter und nicht mehr Entscheidungsträgerin - Empfehlung für andere DDC u. a. sich Zeit nehmen, die eigenen Gefühle auszudrücken und einige körperliche Aufgaben an andere Personen abzugeben |
| Wohlgemuth et al., 2015  USA  A, B, C, D, E, F | Design  Explorative, qualitative Studie  Methode  Halbstrukturierte Interviews  Datenanalyse  Thematische Analyse | Gesamtstichprobe (n=16)  Geschlecht   - Weiblich (n=13) - Männlich (n=3)   Profession   - Pflegefachperson (n=12) - Ärzt:innen (n=3) - Sozialarbeiter:in (n=1)   Arbeitsort/Funktion   - Krankenhaus - Geriatrische Abteilung   Lebenssituation  Keine Angaben  Pflegebeziehung   - Eltern (n=15) - Schwiegereltern (n=1) | - Kleine Stichprobe - Überwiegend Pflegefachpersonen | Schlüsselergebnisse  *Kern- und Unterthemen*   - Vor- und Nachteile der Doppelrolle   A. Vorteile von Fachwissen  B. Herausforderungen und Konflikte aus beruflicher Expertise   - Emotionale Auswirkungen der Doppelrolle   A. Immer auf Abruf  B. Gewollter Stoizismus  C. Anerkennung als das Kind  D. Bürde des Wissens   - Professionelle Auswirkungen der familiären Pflege   A. Einsicht und Empathie  B. Gelernte Lektionen  C. Sich für die richtige Pflege einsetzen  D. Fokussierung auf den Pflegenden  Nächste Schritte/Forschungslücken   - Vergleich von Personen aus der Krankenpflege und anderen Gesundheitsberufen ihre Beteiligung an der Pflege älterer Familienmitglieder angehen - Direkter Vergleich zwischen geriatrischen und nicht-geriatrischen Gesundheitsfachkräften | - Alle entdeckten Phänomene scheinen für alle Disziplinen im Gesundheitswesen zu gelten - Begrenzte Nutzbarkeit, da nicht deutlich wird, welche Aussagen auch für die Pflegenden gelten |

**Tab. 4: Übersicht der eigeschlossenen Reviews und Metaanalysen**

| Authore(n), Land, TK* | Typ von Review oder Meta-Analyse | Eingeschlossene Artikel | Einschlusskriterien | Limita-  tionen | Schlüsselergebnisse der Autoren  Nächste Schritte und Forschungslücken | Schlüsselergebnisse mit Bezug  zu den Forschungsfragen |
| --- | --- | --- | --- | --- | --- | --- |
| Brindley 2018  England  B, E, F | Typ  Qualitative Meta-Ethnography  Methode   - Literaturrecherche in Fachdatenbanken und via Schneeballsystem - Analyse der wörtlichen Zitate der eingeschlossenen Artikel   Datenanalyse  Meta-ethnographisch | Gesamtstichprobe (n=8)  Artikeltypen  Qualitativ (n=8)  Besonderheit  Fünf der eingeschlossenen Artikel waren vom selben kanadischen Forscherteam | Zielgruppe  Beschäftigte im Gesundheitswesen, die auch Familienangehörige pflegen  In den Artikel fokussierte Professionen   - Pflegefachpersonen (n=6) - Andere Professionen (n=2) | Geringe Anzahl an Studien aufgrund der einschränkenden Auswahl der Suchbegriffe | Schlüsselergebnisse  DDC leben gleichzeitig in zwei Welten: in der Familie und im Kreis der Berufskollegen. Es gibt drei verschiedene Arten, wie sie innerhalb dieser Kreise kommunizieren:  a) DDC befinden sich innerhalb beider Kreise: In der Familie, da sie internen Zugang zum Gesundheitssystem haben und so die Familie unterstützen und Fürsprechen können; im Berufskollegenkreis, da sie dort Informationen suchen und erhalten  b) DDC befinden sich innerhalb der Kreise, aber beschränken die Kommunikation: da sie zu viel wissen, teilen sie dieses in der Familie nur noch mit einigen; im Berufskollegenkreis, wenn diese den Druck erhöhen, indem sie ihre Arbeit auf die DDC mit der Begründung abwälzen, sie seien ja vom Fach.  c) In ernsten Situationen, wenn DDC aufgrund ihres Fachwissens ahnen was kommt, z.B. dass der Angehörige sterben wird, ziehen sie sich aus der Familie zurück an den Rand, was sie vereinsamen lässt, sowohl geistig als auch emotional. Im Kreis der Berufskollegen wird dies weiter begrenzt thematisiert.  Nächste Schritte/Forschungslücken  Weitere Untersuchungen zur Ermittlung anderer Phrasen oder Schlüsselwörter zur Erweiterung der Suche | - Je stärker DDC in die Welt der Berufskollegen eintauchen, umso mehr werden sie innerhalb der Familie zu Außenseitern. In dieser Position sind sie mental und emotional allein gelassen. - Manche DDC ändern ihr Verhalten daraufhin gegenüber der Familie, um wieder dazu zu gehören. Andere schaffen es nicht, ihrer professionellen Rolle zu entkommen. - Gegenüber den Berufskollegen müssen DDC immer wieder klarstellen, dass sie nicht als Kollegen, sondern als pflegende Angehörige auf sie zukommen. |
| Detaille et al. 2020  Niederlande  F  Erster Teil des Artikels von Detaille et al., 2020 | Typ  Scoping review  Methode  Systematische Literaturrecherche in Fachdatenbanken  Datenanalyse  Tabellarische Auflistung der Aspekte, die die Forschungsfragen betreffen | Gesamtstichprobe (n=20)  Artikeltypen   - Qualitativ (n=10) - Quantitativ (n=5) - Mixed-Method-Studien (n=3) - Systematisches Review und Meta-Analyse (n=1) - Dissertation (n=1) | Zielgruppe  Pflegefachpersonen mit sekundärem Bildungsabschluss, die (ältere) Erwachsende und/oder Angehörige/Freunde betreuen | - Beschränkung auf englischsprachige Artikel einbezogen - Keine Bewertung der Qualität der Evidenz - Gibt nur einen Überblick über den Stand der vorhandenen Literatur | Schlüsselergebnisse  *Haupt- und Unterthemen der Artikel:*  a) DDC wirkt sich auf das persönliche Leben und die allgemeine Gesundheit aus (Zeitmangel, Verringerung des sozialen Netzwerkes und der Lebensqualität, Erschöpfung, Müdigkeit, Stress, Burnout -> Fehlzeiten)  b) Externe Erwartungen und sozialer Druck als Motivation ein DDC zu werden. Die Grenzen zwischen informeller und formeller Pflege verschwimmen.  c) Selbstmanagement Coping-Strategien durch soziale Unterstützung am Arbeitsplatz und/oder Nutzung bzw. Stärkung der persönlichen Ressourcen  Nächste Schritte/Forschungslücken  Meta-Analysen zur Validierung der vorliegenden Ergebnisse. | Die Beschäftigungsfähigkeit von DDC ist abhängig von den Auswirkungen der eigenen Erwartungen und die der Familie an die eigene Rolle, der erfahrenen Unterstützung am Arbeitsplatz sowie der Selbstmanagementfähigkeiten in der Vereinbarkeit von informeller und formeller Pflege. |
| Giles und Hall 2014  Australia  B, C, D, E, F | Typ  Qualitativer, systematischer Review  Methode  Assessment  Critical Appraisal Skills Program for qualitative studies  Datenanalyse  Thematischer Syntheserahmen | Gesamtstichprobe (n=7)  Artikeltypen  Primäre, qualitative Studien (n=7) | Zielgruppe  Pflegefachpersonen, deren Familienangehörige aufgrund einer schweren Krankheit hospitalisiert wurden | - Begrenzte Anzahl an Studien - Geringe Sorgfalt einiger Einen - Studien nur aus drei Ländern - Einige Studien älter als 10 Jahr | Schlüsselergebnisse  Sechs Eigenschaften/Merkmale kennzeichnen Pflegefachpersonen, deren Angehörige mit einer kritischen Erkrankung ins Krankenhaus eingewiesen wurden:   - *Fachwissen -* kann Ängste und Befürchtungen auslösen, hilft aber auch dabei, unzureichende Pflege zu erkennen oder aussagekräftige und spezialisierte Informationen zu erhalten*.* - *Doppelter Rollenkonflikt -* ständiges Abwägen, wann es besser ist, die Pflegefachperson oder das Familienmitglied zu sein - *konkurrierende Erwartungen -* des Patienten, anderer Familienmitglieder und des Pflegepersonals führen zum Verwischen der Grenzen zwischen informeller und formeller Pflege sowie einem erhöhten Angstlevel - *Aufbau von Beziehungen -* zum Personal, um Informationen zu erhalten, die Angehörigen zu schützen und eine adäquat geleistete Pflege sicherzustellen - ‚*Eingelassen‘ werden* - im Sinne von einbezogen werden, um zu überwachen und zu Fürsprechen - *Gesundheitspflegeeinrichtung (eigene vs. andere*) - spezielle Zugänge und Behandlungen sowie kollegiale Unterstützung erhalten   Nächste Schritte/Forschungslücken  Kenntnisse über Erfahrungen, Herausforderungen und Bedürfnisse der DDC erlangen, um diese effektiv zu unterstützen. | - DDC fühlen sich verpflichtet, andere Familienmitglieder emotional zu unterstützen, wobei sie eigene Ängste und Befürchtungen unterdrücken - DDC müssen ständig Erwartungen und Grenzen aushandeln - DDC fühlen sich isoliert, weil sie niemanden zum Austauschen haben |

**Literaturverzeichnis der eingeschlossenen Studien und zugehörigen Basisartikel**

Anjos, A. P., Ward-Griffin, C. & Leipert, B. (2012). Understanding gendered expectations and exemptions experienced by male double-duty caregivers: A qualitative secondary analysis. *Canadian Journal of Nursing Research*, *44*(3), 104–123.

Baumblatt, G. L., Xu, J., Hanson, G., Masevich, O., Wendel, P., Karavattuveetil, G. & Phillips, J. (2022). The impact on organizations, individuals, and care when nurses are also family caregivers. *Nursing Outlook*, *70*(3), 381–390. <https://doi.org/10.1016/j.outlook.2021.12.001>

Boumans, N. P. & Dorant, E. (2014). Double-duty caregivers: healthcare professionals juggling employment and informal caregiving. A survey on personal health and work experiences. *Journal of Advanced Nursing*, *70*(7), 1604–1615. <https://doi.org/10.1111/jan.12320>

Bray, J., Kelly, E., Hammer, L., Almeida, D., Dearing, J., King, R. Buxton, O. (2013). An Integrative, Multilevel, and Transdisciplinary Research Approach to Challenges of Work, Family, and Health. https://10.3768/rtipress.2013.mr.0024.1303

Carlsson, E., Carlsson, A. A., Prenkert, M. & Svantesson, M. (2016). Ways of understanding being a healthcare professional in the role of family member of a patient admitted to hospital. A phenomenographic study. *International Journal of Nursing Studies*, *53*, 50–60. <https://doi.org/10.1016/j.ijnurstu.2015.10.004>

Cicchelli, L. & McLeod, D. (2012). Lived experiences of nurses as family caregivers in advanced cancer. *Canadian Oncology Nursing Journal*, *22*(1), 53–61. <https://doi.org/10.5737/1181912x2215356>

Clendon, J. & Walker, L. (2017). Nurses as family caregivers - barriers and enablers facing nurses caring for children, parents or both. *Journal of Nursing Management*, *25*(2), 93–101. <https://doi.org/10.1111/jonm.12445>

DePasquale, N., Davis, K. D., Zarit, S. H., Moen, P., Hammer, L. B. & Almeida, D. M. (2016). Combining Formal and Informal Caregiving Roles: The Psychosocial Implications of Double- and Triple-Duty Care. *The Journals of Gerontology / B*, *71*(2), 201–211. <https://doi.org/10.1093/geronb/gbu139>

DePasquale, N., Mogle, J., Zarit, S. H., Okechukwu, C., Kossek, E. E. & Almeida, D. M. (2018). The Family Time Squeeze: Perceived Family Time Adequacy Buffers Work Strain in Certified Nursing Assistants With Multiple Caregiving Roles. *Gerontologist*, *58*(3), 546–555. <https://doi.org/10.1093/geront/gnw191>

Detaille, S. I., Lange, A. de, Engels, J., Pijnappels, M., Hutting, N., Osagie, E. & Reig-Botella, A. (2020). Supporting double duty caregiving and good employment practices in health care within an aging society. *Frontiers in psychology*, *11.* <https://doi.org/10.3389/fpsyg.2020.535353>

Fouto, A. L. R. & Partington, L. (2016). Experiences of healthcare professionals as caregivers of a dying family member: an exploratory study. *International Journal of Palliative Nursing*, *22*(9), 448–453. <https://doi.org/10.12968/ijpn.2016.22.9.448>

Giles, T. M. & Williamson, V. (2015). Torn between dual roles: the experiences of nurse-family members when a loved one is hospitalised in a critical condition. *Journal of Clinical Nursing*, *24*(21-22), 3095–3106. <https://doi.org/10.1111/jocn.12900>

Hansen, J. A. (2016). *The nurse as a family caregiver: Their experience, their story* (1089) [Theses and Dissertation, South Dakota State University]. RIS. <http://openprairie.sdstate.edu/etd/1089>

Häusler, N., Bopp, M. & Hammig, O. (2017). Informal caregiving, work-privacy conflict and burnout among health professionals in Switzerland - a cross-sectional study. *Swiss Medical Weekly*, *147.* <https://doi.org/10.4414/smw.2017.14552>

Jähnke, A., Liebert, Y., Käppeli, A., van Holten, K. & Bischofberger, I. (2017). „Wachsam? Aber sicher!“ Gesundheitsfachpersonen als fachkundige Angehörige und ihre Rolle in der Patientensicherheit. *Pflege*, *30*(6), 375–386. <https://doi.org/10.1024/1012-5302/a000588>

Jones, J. L. (2020). *A different point of view: The lived experiences of registered nurses as family caregivers* (ProQuest 27960230) [Dissertation]. University of Arkansas for Medical Sciences, Arkansas, USA.

Klages, D., East, L., Usher, K. & Jackson, D. (2020a). Health Professionals as Mothers of Adult Children With Schizophrenia. *Qualitative Health Research*, *30*(12), 1807–1820. <https://doi.org/10.1177/1049732320936990>

Klages, D., East, L., Usher, K. & Jackson, D. (2020b). Modes of Informed Caring: Perspectives of Health Professionals Who Are Mothers of Adult Children with Schizophrenia. *Issues in Mental Health Nursing*, *41*(9), 792–798. <https://doi.org/10.1080/01612840.2020.1731890>

Klages, D., East, L., Usher, K. & Jackson, D. (2020c). Post-traumatic growth: Health professionals as mothers of adult children with schizophrenia. *Health Care for Women International*, *41*(8), 916–927. <https://doi.org/10.1080/07399332.2020.1781126>

Mills, J. & Aubeeluck, A. (2006). Nurses’ experiences of caring for their own family members. *British Journal of Nursing*, *15*(3), 160–165. <https://doi.org/10.12968/bjon.2006.15.3.20515>

Quinney, L., Dwyer, T. & Chapman, Y. (2018a). Professional insights from nurses who are carers for family with chronic illness: A phenomenological approach. *Collegian*, *25*(3), 263–269. <https://doi.org/10.1016/j.colegn.2017.09.007>

Quinney, L., Dwyer, T. & Chapman, Y. (2018b). Tensions in the personal world of the nurse family carer: A phenomenological approach. *Nursing Inquiry*, *25*(1), Artikel e12206. <https://doi.org/10.1111/nin.12206>

Salmond, S. W. (2011). When the family member is a nurse: the role and needs of nurse family members during critical illness of a loved one. *Intensive & Critical Care Nursing*, *27*(1), 10–18. <https://doi.org/10.1016/j.iccn.2010.09.002>

Santerre-Theil, A., Brown, T. L., Körner, A. & Loiselle, C. G. (2022). Exploring healthcare professionals’ experiences with informal family cancer caregiving. *Supportive Care in Cancer.* Vorab-Onlinepublikation. <https://doi.org/10.1007/s00520-022-07207-y>

St-Amant, O., Ward-Griffin, C., Brown, J. B., Martin-Matthews, A., Sutherland, N., Keefe, J. & Kerr, M. S. (2014). Professionalizing familial care: Examining nurses’ unpaid family care work. *Advances in Nursing Science*, *37*(2), 117–131. <https://doi.org/10.1097/ANS.0000000000000023>

Ward-Griffin, C. (2004). Nurses as Caregivers of Elderly Relatives: Negotiating Personal and Professional Boundaries. *Canadian Journal of Nursing Research*, *36*(1), 92–114.

Ward-Griffin, C., Brown, J. B., St-Amant, O., Sutherland, N., Martin-Matthews, A., Keefe, J. & Kerr, M. (2015). Nurses Negotiating Professional–Familial Care Boundaries: Striving for Balance Within Double Duty Caregiving. *Journal of Family Nursing*, *21*(1), 57–85. <https://doi.org/10.1177/1074840714562645>

Ward-Griffin, C., Brown, J. B., Vandervoort, A., McNair, S. & Dashnay, I. (2005). Double-Duty Caregiving: Women in the Health Professions. *Canadian Journal on Aging*, *24*(4), 379–394. <https://doi.org/10.1353/cja.2006.0015>

Ward-Griffin, Catherine; Keefe, Janice; Martin-Matthews, Anne; Kerr, Michael; Brown, Judith Belle; Oudshoorn, Abram (2009). Development and validation of the Double Duty Cargiving Scale. *Canadian Journal of Nursing Research, 41*(3), 108-128.

Ward-Griffin, C., St-Amant, O. & Brown, J. B. (2011). Compassion Fatigue Within Double Duty Caregiving: Nurse-Daughters Caring for Elderly Parents. *Online Journal of Issues in Nursing*, *16*(1), 1. <https://doi.org/10.3912/OJIN.Vol16No01Man04>

Wilson, K. B. & Ardoin, K. B. (2013). When Professional and Personal Worlds Meet: Nurse as Daughter. *MEDSURG Nursing*, *22*(3), 192–196.

Wohlgemuth, C. M., Auerbach, H. P. & Parker, V. A. (2015). Advantages and Challenges: The Experience of Geriatrics Health Care Providers as Family Caregivers. *The Gerontologist*, *55*(4), 595–604. <https://doi.org/10.1093/geront/gnt168>

1. Elm E von, Schreiber G, Haupt CC (2019) Methodische Anleitung für Scoping Reviews (JBI-Methodologie). DOI 10.1016/j.zefq.2019.05.004 [↑](#footnote-ref-1)
2. **Behandelte Themenkomplexe**: A = emotionale und persönliche Aspekte, B = Anforderungen und Erwartungen an Double Duty Carer, C = persönliche Bedürfnisse, D = Rollenfindung, E = pflegerisches Fachwissen, F = Interaktion mit Fachpersonen des Gesundheitswesens [↑](#footnote-ref-2)
